# From Bench to Bedside: Governing Health Care Artificial Intelligence (AI) through a “True Lifecycle Approach”

**DOI:** 10.1017/amj.2025.10091

**Published:** 2025-12

**Authors:** Barry Solaiman

**Affiliations:** Affiliate, HMS Center for Bioethics

**Keywords:** Artificial intelligence, Health Care, Governance, Lifecycle, Law, Ethics

## Abstract

This paper addresses the comprehensive regulation of artificial intelligence (“AI”) across its entire lifecycle in the health care sector. It builds on a proposal for a True Lifecycle Approach (“TLA”) to address governance gaps across three phases of AI and expands the framework with detailed practical insights for governing health care AI, drawing on pioneering examples from Qatar, Saudi Arabia, and the United Arab Emirates (“UAE”) as models for global implementation. Beginning with the research and development phase, it highlights the urgent need for robust guidelines and certification processes to ensure that AI technologies are developed in compliance with ethical and safety standards. Moving into the approval stage, the discussion explores how AI systems can be effectively regulated under existing medical device frameworks, emphasizing the need for tailored regulations that consider the unique challenges posed by AI. Finally, the paper delves into the deployment of AI in clinical practice, examining the gaps in current laws and the need for a coherent and consistent regulatory framework that can adapt to AI advancements. The paper argues that the existing legal structures are inadequate, often inconsistent, and fail to address the complexities of AI in health care. It argues for a broader regulatory approach focused on patient safety throughout the AI lifecycle.

## 1. Introduction

This paper details the True Lifecycle Approach (“TLA”) towards governing artificial intelligence (“AI”) in health care. The TLA was first articulated in an earlier perspective paper, and this work builds on that proposal, providing greater details about what the TLA entails in practice for governing health care AI.^1^ The TLA is defined as a comprehensive framework for governing AI in health care, whether the device is used in diagnostics, monitoring, health care administration, or other health care-related applications with a core emphasis on embedding patient protections derived from universal principles of medical law and bioethics.^2^

Providing holistic governance of AI is necessary in health care because existing governance structures do not properly regulate AI.^3^ Where regulations do exist, they largely ignore the patient and fundamental protections afforded to them under principles of medical law and bioethics.^4^ AI is not merely passive, like a blood test kit. AI can be adaptive, autonomous, and be more accurate than practicing physicians.^5^ AI is not imaginary; it is being used in health care systems throughout the world today, making significant impacts.^6^

One survey of hospitals in the United States in 2023 found that sixty-five percent of hospitals used AI.^7^ The United Kingdom’s (“UK”) National Health Service (“NHS”) uses AI to analyze X-ray images (such as mammograms) to support radiologists in making assessments, or helps clinicians read brain scans more quickly.^8^ In China, projections suggested that up to ninety percent of hospitals would use some form of AI by the end of 2025.^9^ In the European Union (“EU”), eighty percent of hospitals are experimenting with AI or attempting to scale the technology across the entire enterprise.^10^ AI is used to support patients in “virtual wards” to receive care at home rather than a hospital.^11^ AI is embedded in wearable devices and is used to monitor patients remotely.^12^ A considerable number of emergency departments throughout England have rolled out AI to identify at-risk patients to provide care at an early stage.^13^

Undoubtedly, there will be more pros than cons for use of AI in health care.^14^ It will unleash exponentially greater benefits for patients. However, given the scale of its deployment, harms and hurdles will occur somewhere, and the law must be equipped to deal with those issues when they arise. Yet, governance frameworks have not been established nor calibrated to meet the scale of the challenge.^15^ Since approximately 2020, a pathway from “AI to Law” has emerged.^16^ Legal scholars have identified key legal issues (primarily arising in tort law) but have found the law to not be fully equipped to deal with the challenges of AI.^17^ At the same time, soft laws have emerged in the form of guidelines underpinned by ethics “principles.” For example, the World Health Organization (“WHO”), the Organization for Economic Development (“OECD”), and others have produced guidelines.^18^ Hard law has only recently emerged in the form of the EU’s AI Act, and the revised product liability directive.^19^

This process of “AI to Law” has resulted in important developments but they miss the mark for health care, for reasons explored in this paper. Namely, existing frameworks do not capture best practices for research and development (“R&D”) of AI. It is unclear what standards (if any) R&D teams are following for devices that will ultimately be used on patients or in health care systems more broadly.^20^ Market approvals processes for AI systems, like those under the Food & Drug Administration (“FDA”), leave out certain devices that make it to market without adequate checks.^21^ Downstream, hospitals are deploying AI without any coherent or clear framework globally.^22^ Most developers and hospitals do not have AI committees to assess the impact of technology internally, but some are making effort, such as in Singapore.^23^ On the legal side, the EU’s AI Act deals with risk mitigation of AI systems, but the governance protections have little relevance to health care.^24^

That is not to say that there have been no advances in scholarship regarding governance frameworks. There are important contributions examined below that certainly advance literature, and more are coming. The first part of this paper argues that where frameworks have been discussed or developed, they are not holistic, often focusing on certain aspects of the AI lifecycle rather than the broad and more complex care machinery that operates around patients. Indeed, another significant omission is the almost complete lack of regard for the most important person in all these considerations — the patient. Humans are at their most vulnerable when faced with a health crisis. A clear and robust system should operate in the background to protect the patient when AI is used, and that system should be communicated to the patient in a simple format, with clear expectations concerning their rights, duties, and redress pathways where things go wrong.

As such, the TLA was proposed as a governance model to bring together the entire AI lifecycle in a holistic manner, embedding health care law throughout and putting patients first.^25^ The TLA consists of three phases of governance at the (1) R&D phase of AI; (2) market approval of AI; and (3) post-AI implementation in practice.^26^ Part two of this paper examines the TLA in detail, and Part three critiques its limitations and highlights areas for future development. The TLA may not be the best proposal for AI governance in health care, but it is the first comprehensive proposal that considers the full lifecycle of AI from R&D to post-implementation deployment with health care law and ethics at its heart.^27^ The TLA may ultimately be rejected as a proposal, but the purpose here is to stir debate and encourage discussion in an area where solutions feel stagnant and discussions repetitive.

It should be noted that the idea for the TLA first emerged when exploring governance models for AI in health care in Qatar, the UAE, and Saudi Arabia.^28^ A separate analysis revealed that each country has had important developments in each area of the AI lifecycle, and it was observed that bringing those separate developments together would represent the TLA that could offer a global governance model for AI in health care.^29^ As such, examples from those countries are primarily given in part two of this paper, whilst being situated within broader developments globally. Examples in the law are also drawn from the largest AI markets in the world (the United States, China, and Europe, particularly in part one of this paper).

## 2. Assessing Existing Frameworks for Health Care AI

### 2.1. The Universality of Medical Law & Ethics

Conceptualizing governance of health care-AI is complex. That process first requires some fundamental soul searching about issues that are hotly debated. *Should* AI even be regulated? The United States perspective might argue that we should only regulate AI to a limited extent and encourage the free market to flourish.^30^ The EU has gone in another direction — creating a behemoth-like regulation via the AI Act.^31^ China opts for another model, characterized by state-centric control linked to state security, social stability, and industrial policy.^32^ There is no correct approach. Much depends on the economic, social, and political context.

Dig deeper into the application of AI to a specific sector like health care, though, and such distinctions in philosophy dissipate (or at least they should). Health care law is underpinned by ethical and legal principles that are largely universal. The interpretation and application of those principles varies but the core principles and approaches are similar. For example, patient autonomy should be respected and operationalized through informed consent.^33^ Of course, autonomy manifests in diverse ways. Informed consent processes in the West are more individualistic and view the patient as a consumer.^34^ In China and the Middle East, informed consent is more familial, with procedures directly involving or devolving authority to family members.^35^ While the manifestation of informed consent processes differs, the core point is that informed consent is a universal principle.

So too are other principles in medical law and ethics. Non-maleficence and beneficence stipulate that providers should not harm patients and act in their best interests.^36^ These principles manifest through the law via the duty of care and malpractice liability under torts.^37^ There are “no-fault” liability schemes, such as in New Zealand and the Nordic countries but those are in the minority, and in any case, those schemes exist to compensate for wrongs even if no one is held to be liable.^38^

The confidentiality of patients must be upheld to preserve trust in the doctor-patient relationship, and for many other important reasons. Such protections are found in a plethora of sources, such as laws, guidance documents, data protection standards, and more. For example, the General Data Protection Regulation (GDPR) treats health data as data of a “special category” with regulations from other countries also treating health data in a similar manner.^39^ That designation gives such data extra legal protection.^40^ Confidentiality is addressed in case law. For example, in England, the courts have long recognized that “there is an abiding obligation of confidentiality as between doctor and patient.”^41^ In China, statute obligates that “medical and healthcare institutions, and medical and healthcare professionals shall … protect patient[]” privacy.^42^

There are also obligations that apply internationally through treaties. The International Covenant on Economic, Social and Cultural Rights (“ICESCR”) recognizes the “right of everyone to the enjoyment of the highest attainable standard of physical and mental health.”^43^ That right to health should be exercised “without discrimination of any kind as to race, colour, sex, language, religion, political or other opinion, national or social origin, property, birth or other status.”^44^ In other words, health care should be delivered equitably and without discrimination.^45^

Underpinning all the above is that there should be respect for human dignity, as recognized under the Universal Declaration on Bioethics and Human Rights (from UNESCO).^46^ “The interests and welfare of the individual should have priority over the sole interest of science and society.”^47^

Health care presents a universally consistent legal and ethical foundation for the protection of patients. The extent to which governments may seek to regulate AI will always run up against these expectations when AI systems are intended for use in health care; it is for this reason that any governance framework must encapsulate those universal protections and expectations.

Another layer that is important is the existing national regulatory processes for medical device approvals to market. The FDA is the most well-known, but many such agencies exist, such as the Ministry of Food and Drug Safety (“MFDS”) in South Korea, or the Saudi Food and Drug Authority (“SFDA”), discussed later in this paper. These regulators have separate technical rules to determine whether medical devices can be brought to market.^48^ The WHO has a global benchmarking tool (“GBT”) for evaluating national regulatory systems of medical devices, but very few countries meet the highest standards set by the tool, likely because the majority of countries do not have a ‘mature” infrastructure for medicine and vaccines.^49^

### 2.2. Why AI Presents New Challenges in Health Care

Given that medical law and ethics principles are largely universal, and that there are at least *some* regulators, like the FDA, that already have processes for approving medical devices to market, it may be argued that the existing governance paradigm is sufficient (with some adaptations) when applied to AI devices used in health care. That proposition is true to an extent, but AI stretches the law further than its design.

AI devices can make decisions and interpret information without human input, which raises questions about legal accountability in medical decision-making.^50^ AI devices have “adaptive” algorithms that learn and adapt over time to the data processed.^51^ Medical device regulations have typically covered “locked” algorithms, meaning that the same result is produced from the same input every time it is used.^52^ It is harder to regulate adaptive algorithms because the device may produce different outputs than expected at the time of regulatory approval.^53^ The law has not been equipped to deal with that challenge.

Further, since adaptive algorithms can “drift” over time without any deliberate changes from the manufacturer,^54^ the standard of care may become more contested where AI proves to outperform clinicians across a broad range of clinical practice. If that becomes the case, then the courts will be asked to decide whether the standard of care itself should be changed to align with AI outputs.^55^ In other words, could we shift to a system where AI recommendations are the standard of care?

Explainability presents another challenge.^56^ Informed consent requires that patients are able to understand all relevant information about diagnostic processes or proposed treatments for their care, and then voluntarily agree to that treatment.^57^ To understand all relevant information, the health care professional should be able to provide an explanation to the patient in terms they can understand.^58^ Challenges arise if AI is used to provide treatment recommendations, but AI cannot provide an explanation for why it made a specific recommendation.^59^ Even if AI can provide an explanation, it cannot be trusted because AI often “hallucinates” and invents evidence to support its recommendations.^60^ If patients rely on such hallucinations to make a decision, then the threshold for proper informed consent cannot be met because their decision may be based on a falsehood. This is not an imagined concern. AI has already made harmful recommendations that may have *increased* the risk of suicide in those following its advice.^61^

Of course, there is case law about whether a particular medical interaction involved adequate informed consent, but those cases involve investigations about who said what, and one can ask the doctor why they made a particular recommendation.^62^ For AI, even computer scientists can struggle to understand *why* AI made a particular recommendation.^63^

Liability becomes further muddied in those waters. For example, there is the “learned intermediary” principle, where a manufacturer has a duty to warn a patient about the risks of a medical device, by providing adequate warnings to the prescribing doctor instead of to the patient.^64^ Price has queried whether that doctrine must “bow to the recognition that doctors cannot understand all the technologies they use or the choices such technologies help them make when they are not provided the needed and/or necessary information?”^65^ What if a clinician is overruled by AI? How is causation established in this arena of diffused responsibility when the traditional patient-doctor accountability loop is altered?

Data vulnerabilities present a double-edged legal challenge in AI. Health care data could be centralized (for example, within a hospital system), and AI could analyze data within that system only.^66^ However, that single point of entry creates vulnerabilities for adversarial attacks.^67^ At the same time, systems could be decentralized, with data stored in multiple locations across borders. AI processing could even be undertaken in the cloud. But this latter approach presents legal compliance issues.^68^ Many companies are unclear about their obligations in this landscape.^69^

Bias and inequity also present new challenges. Algorithms may be trained on inherently biased data, which could lead to useless or harmful outputs.^70^ The law has not risen to this challenge. A major query is how bias and discrimination can be dealt with under equality laws.^71^ This also invites the question about whether product liability laws or data protection laws are also applicable. There is also the potential for conflict between different areas of law. Manufacturers can refuse to disclose their algorithms for inspection under intellectual property law.^72^ These tensions have played out in recent EU case law: the Court of Justice of the European Union (“CJEU”) held that the courts may have to balance trade secret protections against a data subject’s rights to access “meaningful information” about the logic involved in the automated decision of an algorithm.^73^ Indeed, much remains unsettled in this space.

Finally, there is simply the issue of over-reliance on AI systems by medical professionals subordinating their own clinical expertise and decision-making.^74^ AI models that are over-generalized may not be useful to a specific patient, but the clinician may nonetheless follow its recommendation.

Ultimately, it is inaccurate to argue that AI does not present new challenges for the patient. Indeed, AI presents new and pressing challenges that the law must deal with.

### 2.3. Are current governance frameworks sufficient?

There has been some important framing scholarship for identifying the legal issues noted above. For example, Gerke, Minssen, and Cohen highlighted legal and ethical challenges in the field in 2020.^75^ They noted the ethical challenges of informed consent, safety and transparency, algorithmic fairness and bias, and data privacy.^76^ Legal challenges highlighted were safety and effectiveness, liability, data protection and privacy, cybersecurity, and intellectual property.^77^ Solaiman and Cohen’s book on “Health, AI and the Law,” included more detailed analyses of the legal issues, which they framed as algorithmic discrimination and health equity, data protection, data security, liability, informed consent, and intellectual property.^78^ Solaiman and Cohen’s book also charts the governance developments globally, revealing that very little progress has been made on the underlying legal issues for health care.^79^

However, there have been developments in three areas explored in the passages below that are consistently characterized or premised on a total lifecycle approach. First, at the global governance level through the WHO. Second, through the development of medical device regulations in some countries like the United States, Singapore, South Korea, China, and other countries. Third, through the passage of the EU’s AI Act. The main developments are outlined below and contrasted with the TLA proposed later in this paper.

#### 2.3.1. The WHO and other bodies

In 2023, the WHO published *Regulatory Considerations on Artificial Intelligence for Health.* ^80^ The publication set out six broad topic areas: documentation and transparency, risk management, intended use and validation, data quality, privacy and data protection, and engagement and collaboration.^81^ Central to the document was the Total Product Life Cycle (“TPLC”). The WHO emphasized:

A [TPLC] should be considered throughout all phases in the life of an AI system, namely: pre-market development management, post-market surveillance and change management. In addition, it is essential to consider a risk management approach that addresses risks associated with AI systems, such as cybersecurity threats and vulnerabilities, underfitting, algorithmic bias etc.^82^

The WHO seeks to promote this approach with documentation and oversight that spans pre-market, deployment, and post-market phases of AI.^83^ The focus is on holistic risk management through the use of a quality management system, development practices for AI systems, cybersecurity, performance evaluation, and more.^84^ In this regard, the WHO’s recommendations are comprehensive, with a global outlook that aims to build shared regulatory expectations.

While this approach is important and detailed, it remains primarily technical and regulatory in nature. The recommendations do not embed accountability and redress mechanisms focused on patients, nor do they explicitly integrate medical law doctrines, or local and ethical considerations. By contrast, the TLA developed in this paper seeks to develop a more patient-centric governance framework.

#### 2.3.2. The FDA’s Medical Device Regulations & Related Agencies

Aside from the WHO’s framework, medical device regulators have also developed rules to account for AI. The United States FDA is the most prominent example with its TPLC approach that is “focused on device oversight throughout the product’s life cycle — from device design and development to real-world use of the device.”^85^ The TPLC seeks to promote transparency, efficiency, and agility of FDA oversight by making information sharing between regulators easier, compressing levels of review, and helping employees develop a deeper view of device safety.^86^ It is emphasized that the suite of guidance documents produced by the FDA are of great import and there is some crossover in underlying philosophy between the TLA, but the focus is different for reasons unpacked below.

In recent years, the FDA has adapted the TPLC to AI-enabled devices. In 2025, it issued draft guidance on AI-enabled device software functions.^87^ That guidance includes detailed recommendations on premarket submissions, with requirements on quality systems, risk assessments, data management, and performance validation.^88^ It also discusses post market monitoring plans to detect data drift and to respond to safety issues once in use in the real world.^89^ In 2021, the FDA also issued the Good Machine Learning Practice (“GMLP”) principles alongside Health Canada and the UK’s Medicines and Healthcare products Regulatory Agency (“MHRA”).^90^ The GMLP emphasize the importance of leveraging multi-disciplinary expertise throughout the TPLC, implementing good software engineering and security practices, that clinical study participants and data sets are representative of the intended patient population, and that deployed models are monitored for performance, among others.^91^

The focus of the GMLP on such issues squarely confronts the concern about bias and discrimination in the use of AI in health care. The FDA defines AI bias as follows:

AI bias is a potential tendency to produce incorrect results in a systematic, but sometimes unforeseeable way, which can impact safety and effectiveness of the device within all or a subset of the intended use population (e.g., different healthcare settings, different input devices, sex, age, etc.).^92^

Thus, in monitoring a device post-deployment, appropriate controls should be in place to manage risks of overfitting, unintended biases, or degradation of the model that may impact the safety and performance of the model.^93^ The FDA recommends that developers use “unbiased, representative training data for models” to avoid “perpetuating biases or idiosyncrasies from the data itself.”^94^ While acknowledging that mitigating bias “may be difficult to eliminate completely” it recommends that, as a starting point, “validation data sufficiently represents the intended use (target) population of a medical device.”^95^

In 2024, the same agencies also issued transparency principles for machine learning-enabled devices.^96^ The principles are relevant to those who use the devices such as health care professionals, patients, and caregivers.^97^ They note that “transparency is essential to patient-centered care and for the safety and effectiveness of a device” and that “logic and explainability are aspects of transparency.”^98^ They recommend clear communication of the intended use, limitations, and performance to end users of AI devices.^99^ The transparency principles allude to the informed consent process but do not address it directly. For example, they note that “effective transparency that helps parties make informed decisions can help control risks.”^100^ However, this is never explicitly tied to principles of health care law and ethics, and the expectations and protections that patients may have in this regard.

Finally, in 2024, the same agencies developed quite innovative policies on Predetermined Change Control Plans (“PCCP”).^101^ PCCPs are plans proposed by manufacturers of AI medical devices that specify planned modifications to devices, protocols for implementing and controlling those modifications, and include an assessment of the impact of modifications.^102^ The PCCP principles are particularly focused on developing Principle 10 in the prior GMLP on monitoring for performance and managing re-training risks for deployed models.^103^ Principle 10 stipulates that deployed models have the capability to be monitored in the “real world” with a focus on maintained or improved safety and performance.^104^ In this regard, five guiding principles are developed.

First, any changes that a manufacturer intends to implement should be “focused and bounded.”^105^ Changes should be limited to modifications within the intended use or intended purpose of the original device, and there should be plans in place to “safely modify the device within the bounds of the PCCP.”^106^ Second, the intent, design, and implementation of a PCCP should be driven by a risk-based approach.^107^ Doing so strengthens the value and reliability of a PCCP.^108^ Third, PCCPs should be evidence-based, following scientifically and clinically justified methods and metrics used to measure device performance.^109^ Fourth, PCCPs should have ongoing transparency to users and stakeholders. This helps those users to stay aware of the device’s performance before and after changes are implemented, which is crucial for monitoring, detection, and providing an appropriate response to deviations in device performance.^110^ Fifth, the PCCP should be created from a TPLC perspective.

The guiding principles concerning PCCPs are by no means settled. The agencies intend that their principles lay a foundation for PCCPs and encourage international harmonization, and they encourage ongoing feedback in this space because PCCPs may be developed and implemented in different ways in different jurisdictions.^111^

The collection of guidance documents released in recent years are reflected in the 2025 draft guidance noted above.^112^ For example, that guidance states that “manufacturers should have a postmarket performance monitoring plan to help identify and respond to changes in performance in a postmarket setting.”^113^ In doing so, manufacturers will “reduce uncertainty” and “support the FDA’s evaluation of risk controls.”^114^ Such continuous monitoring is especially important because “models are highly dependent on the characteristics of data used to train them, and as such, their performance can be particularly sensitive to changes in data inputs.”^115^ Existing obligations for monitoring devices remain, such as reporting serious injuries or malfunctions.^116^ The distinction for AI is that the FDA emphasized continuous monitoring, which is an extension of the TPLC’s philosophy that governance does not end with device approval but is an ongoing process post-approval.^117^ Embedded within that paradigm is a more concerted focus on issues that particularly matter in the AI space that the law has yet to properly grapple with, such as bias and discrimination.

Despite these aims, the FDA’s post-market surveillance framework for AI devices has been strongly criticized. The FDA has a repository for post-market surveillance reports called the Manufacturer and User Facility Device Experience (“MAUDE”) database.^118^ This is part of the reactive element of the governance framework meant to ensure that adverse events and malfunctions are submitted by manufacturers and others. It has been argued that this system is “insufficient for properly assessing the safety and effectiveness of AI/ML devices.”^119^ Scholars have assessed how well equipped the long-standing system is at “capturing the kinds of issues and problems that are especially likely to arise from AI/ML devices.”^120^

For example, more than ninety percent of AI/ML device reports were labeled as “malfunctions” but the information given does little to reveal the true severity of the problems.^121^ This, it was noted, was “emblematic of more general issues” as to why the reporting structure is “not fit for purpose for evaluating AI/ML devices.”^122^ Those issues include the sheer extent of missing data that makes it difficult to study the safety of AI/ML devices, inadequate event classification which reveals a disconnect between the challenges arising in practice and the constraints of the categorization in the system, and the severity of the risk being unknown.^123^

It is not only structural problems that are of concern. Conceptually, the FDA’s TPLC is designed to deal with approving medical devices, with a focus on monitoring safety and effectiveness throughout the device’s life cycle.^124^ It will be seen below how the TLA is a broader and deeper framework that seeks to consistently embed patient protections arising from medical law and ethics.

#### 2.3.3. The EU’s AI Act

The AI Act should be understood in a quite different context to the FDA guidelines. The most significant difference is that it is binding “hard law,” as opposed to the FDA guidance documents which are technically non-binding, and remain under consultation.^125^ The AI Act is also not focused on any sector but is a broad regulatory scheme premised on classifying the risk of AI systems and through that classification imposing certain requirements on manufacturers and others before permitting those devices to market.^126^ Nevertheless, the AI Act is premised on promoting the uptake of AI while also ensuring a “high level of protection of health, safety, [and] fundamental rights as enshrined in the Charter of Fundamental Rights of the European Union.”^127^ That articulation does broadly bring health within the explicit aims of the Act.

The Act classifies AI based on risk. AI devices such as social scoring systems are deemed to pose an unacceptable risk and should be prohibited from the market.^128^ AI systems that pose a minimal risk, such as wellness apps on an app store are unlikely to be captured by the Regulation.^129^ Most medical devices will fall under the “high risk” category and will be subject to certain checks before being permitted to market.^130^ The Act incorporates the Medical Devices Regulation (“MDR”)^131^ and the in vitro Diagnostic Medical Devices Regulation (“IVDR”)^132^ into its annexes.^133^ Manufacturers must comply with safety and other requirements of the MDR or IVDR, and also the specific requirements of the AI Act where AI is incorporated into the device.^134^

There is a “conformity assessment” procedure for demonstrating whether the requirements for high-risk AI systems have been fulfilled.^135^ Conformity assessments are rather convoluted processes involving a third party to obtain a “CE” mark.^136^ The assessments are already required under the MDR/IVDR and have been criticized for providing weak protections for human health and safety.^137^ The process ultimately amounts to self-certification, with some noting that “it is seriously questionable whether reliance on self-certification provides meaningful legal assurance that the requirements to obtain a CE mark in relation to high-risk AI systems are properly met.”^138^

Aside from that specific conformity assessment procedure, the AI Act has some structural similarities to the FDA approach. It requires that a risk-management system should be implemented that is continuous, iterative, and runs “throughout the entire lifecycle of a high-risk AI system.”^139^ It emphasizes post-market monitoring of devices to identify any need to immediately apply corrective or preventative actions.^140^ A post-market monitoring system should actively and systematically collect, document, and analyze data to evaluate such compliance.^141^

The AI Act also covers biases under its data requirements, requiring that training, validation, and data sets shall be subject to practices that include an “examination in view of possible biases that are likely to affect the health and safety of persons, have a negative impact on fundamental rights or lead to discrimination prohibited under Union law, especially where data outputs influence inputs for future operations.”^142^

Appropriate measures must be taken to “detect, prevent, and mitigate” those possible biases.^143^ In this manner, there are similarities to other lifecycle approaches but the explicit focus on health care is limited. Joint guidance issued by the EU’s Medical Device Coordination Group (“MDCG”) and the AI Board (an advisory body created by the AI Act), notes that the MDR/IVDR address general medical device software risks but “they do not explicitly address risks specific to AI systems. The AIA complements the MDR/IVDR by introducing requirements to address hazards and risks for health, safety[,] and fundamental rights specific to AI systems.”^144^

The guidance emphasizes that the obligations under the AI Act complement the other regimes but are separate from them.^145^ Compliance with one set of requirements does not equate to compliance with the other and both schemes must be complied with, but it is likely that compliance tasks will be integrated by developers in such a way to avoid duplicity of efforts, something that is explicitly encouraged.^146^

Despite some areas of structural overlap between the AI Act and the FDA’s TPLC, they are ultimately different in design. The AI Act is a comprehensive binding governance model that defines rules, responsibilities, and creates new institutions with oversight. The FDA’s TPLC is more of a conceptual approach within existing legal frameworks. Importantly for the TLA proposed in this paper, both the AI Act and TPLC have a lifecycle approach and focus (to a certain extent) on ethical safeguards through their focus on data bias (for example), but both omit other patient-centric elements.^147^ Neither system directly or explicitly incorporates the suite of health care law concerns identified in the scholarship in a comprehensive and consistent manner nor offer realistically actionable redress mechanisms for patients if things go wrong.

The AI Act has already been criticized elsewhere as being “flawed.”^148^ The high-risk category has been labeled as “deficient” for patients because it limits their power of redress.^149^ Individuals have the right to complain about infringements in the Regulation to the “market surveillance authority” but this ultimately bears little connection to patient’s rights in the health care system.^150^ Indeed, Ebers argues that “one of the most crucial points” about the AI Act is that it provides “limited individual rights” beyond the ability to lodge a complaint or the right to an explanation of individual decision-making.^151^

One of the fundamental aims of the AI Act was to build trust but it has been argued that it “falls short of connecting its premise on trust with its risk-based approach.”^152^ Indeed, the rubrics discussed above are not sufficient for ensuring that patients are protected from potential AI harms.

#### 2.3.4. Appraising the Existing Lifecycle Approaches

There is a growing body of research that underscores how those governance frameworks are fragmented and incomplete despite being a step in the right direction. Those gaps mean that patients may be exposed to harms, potentially undermining the very trust those frameworks seek to build.

Regarding the EU regime, Ebers notes that “neither the EU’s Medical Device Regulation nor its [AI] Act adequately address the risks to patient health and safety that arise in many situations where AI systems are used in the healthcare and nursing sector.”^153^ The MDR only applies to tools that are explicitly marketed with a medical purpose, and it is concerning that labeling a product as general “wellness” would avoid oversight.^154^ Ebers argues this is “problematic” given that there are no other rules to protect users from such products.^155^ Further, even where the MDR applies, it only covers the manufacturer to ensure safety but omits health care professionals and others.^156^ While the AI Act does create some obligations, it “does not establish duties to individuals affected by any AI system (patients, care recipients or others).”^157^

The concerns are not limited to the lack of coverage for individual rights but extend to fundamental flaws in the regulatory scheme itself as intended. There are concerns from manufacturers that such regulations will place an undue burden financially and may pose an existential threat to their business.^158^ There are already not enough notified bodies to undertake third party conformity assessments under the MDR, causing delays and backlogs for devices to come to market.^159^ This will become worse under the AI Act given that more products will be captured by its scheme.

Scholars on all fronts have argued that patient-centered governance of AI should engage with fundamental rights more directly than existing schemes. Ho has praised the “adoption of a total device/product-specific lifecycle approach (rather than one that is point-in-time)” as being “more collaborative and participatory in nature, and anticipatory in character.”^160^ However, he argues that even such lifecycle approaches should be grounded in an internationally recognized “human right to science” (HRS).^161^ The HRS is a rights-based approach that obligates regulators to ensure that everyone can “participate in and enjoy the benefits of scientific and technological progress.”^162^ In this manner, regulation should not merely be about risk mitigation but should also encompass public participation and equitable access to new innovations. Ho argues that by applying a HRS, patients can benefit from continuous improvements in AI devices.^163^ A lifecycle regulatory model incorporating HRS would be both participatory (by involving stakeholders beyond manufacturers and regulators), and anticipatory by adapting governance as AI evolves.^164^

Ho rightly notes that traditional static forms of medical device regulations fall short of HRS goals because “patients will not have the opportunity to benefit from” AI improvements.^165^ Scientists will also “not be able to push forward AI science” because AI cannot be limited to trials undertaken in strictly “controlled conditions.”^166^ The rights based approach he argues for is aimed at continuously balancing the interests of innovation with safeguarding patients.^167^ Nevertheless, he also cautions against lifecycle governance approaches becoming overly bureaucratic and intrusive on scientific activities.^168^ He notes that HRS provides “instructive guidance” in that the “enjoyment of the benefits of science” is contingent on protecting the freedom that is indispensable for research and the rights of developers, even where accountability is required.^169^ Ultimately, Ho’s framework (while conceptual) goes further than the FDA’s risk based model, or the EU’s AI Act — with its abstract pledge to uphold fundamental rights and build trust in AI — by proposing to make such rights a fundamental part of the process.

Elsewhere, Cohen et al. have argued that patients should be empowered through the creation of an appeals process for AI decisions.^170^ Looking to legal appeals processes, they suggest that there should be a structured way to appeal an important AI decision, such as the prioritization of care for one patient over another, or a diagnosis that the patient or doctor doubts.^171^ Human expert judges could be included in those appeals processes to review AI decisions. A human reviewer could consider case specific information and provide more nuanced clinical, moral, or legal reasoning — serving as an error correction check on AI.^172^ This approach resonates with care that is patient-centered because patients could seek a human review of an AI decision. While the EU’s AI Act requires human oversight, that does not translate into a specific right for patients to have a review.

The call to follow a true lifecycle mindset is echoed in other scholarship going as far back as 2020. For example, Gerke et al. argued that regulators like the FDA need to “widen their scope” for assessing systems.^173^ The authors noted that the shift in perspective from a product to a system view is necessary to maximize the safety and efficacy of AI in health care.^174^ To achieve this through transitional ‘first steps’ (rather than a full system model) they proposed examining how healthcare professionals react to AI and require training.^175^ The ‘full system approval’ could be expanded to include workflow integrations, hospital authorizations (specific to the particular hospital), liability considerations, insurance, and the ongoing reevaluation of algorithms.^176^ Whilst the FDA does not regulate the practice of medicine, the authors argued that it could require AI developers to set up training programs for their product.^177^ They also argued for ongoing system monitoring, periodic retraining, software and usage inspections, and reviews of aggregate usage statistics (suggestions that are beginning to form part of the post-market monitoring thinking today).^178^ Thus, even in 2020, these ideas illustrated how a regulator like the FDA might operationalize a lifecycle approach by going beyond the pre-market evaluation of devices and towards the ongoing interactions between AI and its users post-market. Those recommendations align with Ho’s more recent HRS arguments.^179^ They converge on the idea that regulating health care AI requires continuous governance.

Hassan et al. have also argued for an “adoption-centered governance framework” that “covers the entire cycle of an AI system, from concept through to sustainability.”^180^ That framework is premised on the creation of a governance committee, which applies “gated mechanisms” at various stages of AI development.^181^ This approach incorporates an analysis of concerns surrounding “bias, equity, transparency, ethics (of AI), explainability, data handling, and safety.”^182^ While their research does not discuss the FDA or the law, the approach somewhat reflects the FDA’s TPLC, but appears to include more ethical checks.^183^

Ultimately, the literature is coalescing around a lifecycle approach towards governing AI that focuses on the patient, the continuous evaluation of AI systems, and the adaptability of design following market deployment. The TLA broadly captures these aims as will be seen below. Like Ho’s HRS framework, the TLA requires ongoing engagement and participation from all stakeholders, especially the patient.^184^ And, like Gerke et al.’s system approach, the TLA emphasizes the monitoring of AI in real world clinical settings while updating that oversight as the systems evolve.^185^ However, it also differs from those frameworks. Thus, Ho’s framework focuses on high-level human rights obligations, but the TLA translates similar values into specific requirements and checkpoints spanning the AI lifecycle.^186^

The next section details how the TLA can build on these insights in practice. This proposal may not solve every problem, but if it at least spurs fresh discussions, we can come a step closer to resolving the health care AI governance puzzle.

## 3. The TLA

**Figure 1.**
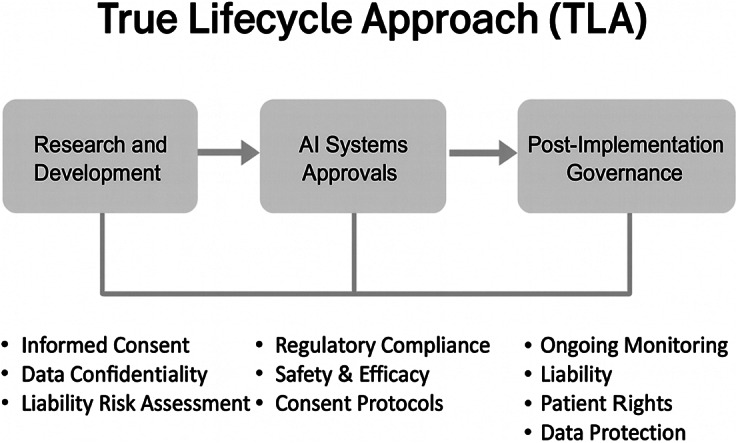
The TLA^194^

The concept of the TLA builds on existing research. In 2024, Solaiman, Bashir, and Dieng outlined how health care AI is governed in Qatar, Saudi Arabia, and the UAE (with a focus on Dubai and Abu Dhabi).^187^ Their research revealed how AI is governed in those countries and highlighted that each jurisdiction was governing different aspects of AI.^188^ In Qatar, there was the creation of the *Research Guidelines for Healthcare AI Development* by Solaiman et al. with advisory support of the Qatar Ministry of Public Heath, which were finally published in 2025.^189^ Those non-binding guidelines cover the research and development stage of health care AI.^190^ In 2024, Solaiman also undertook a detailed analysis of the Saudi Food and Drug Authority’s Guidance on Artificial Intelligence (AI) and Machine Learning (“ML”) technologies based Medical Devices.”^191^ That guidance covers the approval of medical AI devices to market, and goes beyond efforts made by other regulators.^192^ Finally, in 2025, Solaiman et al. proposed the TLA for governing health care AI that synthesized earlier research.^193^ [Fn fn194] This section provides are more detailed examination of the TLA, building on previous research. The term *True* in “True Life Cycle Approach” is used because other frameworks do not fully capture the AI lifecycle, which focus instead on certain stages of its use or development. The TLA is designed to cover all stages of AI as can be seen in Figure 1 below:

### 3.1. R&D & the Qatar Example

Qatar’s *Research Guidelines for Healthcare AI Development* arose from a multidisciplinary three-year research grant funded by Hamad Bin Khalifa University (“HBKU”) led by the author.^195^ They are a pioneering attempt to regulate AI at the research and development stage, which has often been overlooked by other frameworks.^196^ To ensure a seamless discussion with stakeholders, Qatar’s Ministry of Public Health (“MOPH”) were included as official advisers to the grant and involved at all major stages.^197^ The team consisted of scholars and experts from HBKU’s College of Law, College of Health, the Qatar Biomedical Research Institute, the College of Science and Engineering, the College of Islamic Studies, and the Qatar Genome Program (now, the Qatar Precision Health Institute).^198^ Stakeholders external to the grant but located in Qatar were also consulted in-depth at expert roundtables. The aim was to develop guidelines grounded in the experiences and expertise of those in health, law and bioethics with a focus on local norms. The guidelines were, therefore, adapted in places to the local context but the overarching framework was designed to be applicable in other countries with adaptations.^199^

The guidelines provide non-binding best practices for researchers creating AI systems for use in the health care sector broadly defined.^200^ They embed legal and ethical principles from the very outset of AI innovation across three stages: (1) development; (2) external validation; (3) deployment. Across all stages, the principles of fairness, accountability, and transparency are emphasized but adapted to the local Qatar context. For example, “fairness” in the guidelines is broadly a concern about the underrepresentation of data from the Middle East used to train AI systems.^201^ The principle of accountability encompasses Islamic Bioethics perspectives that emphasize human rather than machine responsibility.^202^ This approach seeks to support existing priorities at the national level concerning health care research that should empower patients, empower researchers and medical professionals, advance public health goals, facilitate doctor-patient relations, and develop digital tools that are ethically sound.^203^ Examining the national context is crucial when developing such guidelines to ensure that priorities are aligned across existing ecosystems.

The principles and underlying aims carry through into the guidelines themselves that serve as a detailed technical checklist for researchers to complete to have a detailed record of following best practices.^204^ They explicitly address Qatar’s regulatory and cultural context. For example, one section of the guidelines asks researchers to address data limitations and biases by recording the following information in Table 1.

**Table 1.** Extract From Qatar Research Guidelines for Health Care AI Development:^205^

Data Limitations & Biases: State the method used to determine which data was incorporated into the model Confirm what factors are being examined by the AI system and justify those factors. Factors include, but are not limited to, data on gender, race, health, culture, demographic, and phenotype. Disclose any biases that the model could have possibly encoded. Include the following: Data-sensitivity of the model Whether the model is intended to be used to provide information about human health Consideration for majority/minority groups Bias caused by under-representation Identify the potential risks and harms in the model to any individuals, groups, or demographics Determine whether alternatives to sensitive or identifiable data can be used and outline any steps taken to minimize data biases when developing the AI system concerning: Citizenship Residency Religion Ethnicity Age Race Gender Other traits relevant to Qatar and the Arab region. State transparently any limitations to the model if biases cannot be removed

In this regard, Qatar’s diverse population is considered at the outset because AI researchers must account for under-represented groups in datasets (for example, non-Arabic or English speakers) to prevent bias. By asking researchers to consider issues such as ethnicity, citizenship, and language, the aim is to preempt AI biases before the device has been deployed for use. This aligns the national strategies noted above that encourage the empowerment of patients and developing digital tools that are ethically sound.

Elsewhere, the guidelines state that researchers should state “whether data processing used in the research complies with data protection laws in Qatar”.[Fn fn205] ^206^ In particular, compliance with Law No. 13 of 2016 on Privacy and Protection of Personal Data and accompanying guidelines for regulated entities.^207^ In essence, the rules are encouraging legal and ethical best practices during development rather than trying to resolve issues after the fact once system has been deployed. The guidelines encourage researchers to keep comprehensive documentation of how the AI model was developed, its intended use on patient populations, and the chosen accuracy thresholds for the algorithm, and much more across its approximately fifty pages.^208^

Researchers do not have to satisfy every requirement, but this level of operational detail would certainly help to address any legal compliance questions later. One provision even encourages the researchers to state “any liaison with the Ministry of Public Health or any other relevant regulatory entity on any aspect of the algorithm’s development and deployment.”^209^ These are not merely abstract principles but are concrete tasks that teams can incorporate into their project management and research protocols.

In this regard, these guidelines can help fill a critical gap in existing governance approaches. Current approaches, such as the FDA or EU address device approvals or post-market monitoring but offer little at the research stage where design choices will be most consequential. By integrating such guidelines into a TLA governance framework, this approach could offer a baseline for best practices for ethical and legal AI design with the local context in mind.

A key question that remains outstanding is how to formally integrate the guidelines into research oversight. The grant project envisaged a certification process.^210^ Researchers can complete the checklist, submit it to a committee to review the AI system for compliance, and receive certification as a mark of credibility for the project.^211^ A mock certification website was developed for researchers to complete the checklists and keep a record.^212^ This worked from a technical standpoint but did not resolve the overarching question of whether a certification process is the best method. An alternative method would be to integrate the guidelines somewhere into Institutional Review Board (IRB) processes. For example, an IRB might require researchers to append the completed AI guideline checklist to their application, demonstrating due diligence. However, ultimately, it was determined that the guidelines should exist to complement IRB processes rather than replace them, a prudent approach since the optimal way to integrate these AI guidelines is still unresolved.^213^

Ideally, a separate expert committee should exist through a Ministry approved body, or department within the Ministry of Public Health that can review submissions and provide certification. The committee would be analogous to the IRB but with a specific focus on AI ethics and safety. While not binding, the MOPH could recognize such certification and provide certain legal safeguards for purchasers of certified AI systems in health. For example, certification could act as a defense against liability claims for harms caused by the AI system — perhaps a no-fault scheme could operate where a certified AI system that followed existing best practices causes harm. Another approach might be for the MOPH to require certification as a pre-requisite for government funded health AI projects or for access to national health data.

Regardless of the path chosen, the underlying point is that health care AI reviews could be institutionalized with a mandate for researchers to undergo such assessments. It would require finding experts and forming such a committee (no easy task). But it could “support smoother transitions into the approval and post-implementation stages of the TLA.”^214^ As noted in earlier research, in addressing “these considerations early, researchers reduce the risk of delays or rejections during approval while ensuring systems are better positioned to meet the requirements of medical device regulators.”^215^

Overall, Qatar’s guidelines demonstrate a strong proof of concept for the first phase of the TLA. Early-stage governance at the research phase can be both comprehensive and practical, covering a plethora of legal and ethical issues. The challenge ahead lies in formalizing these practices. Qatar’s early-stage AI guidelines illustrate that rigorous R&D governance is feasible in practice and adaptable beyond the Qatari context. The core design — embedding law and ethics into technical checklists — can be localized to different legal systems, including those without the same centralized health care oversight.

### 3.2. Market Approval & the Saudi Example

An AI medical device should require some form of regulatory approval before going to market in a country. The FDA is clearly at the forefront of discussions in the AI space but not all countries have the equivalent of the FDA. Another pioneer is Saudi Arabia through the Saudi Food & Drug Authority (“SFDA”). In 2022, the SFDA issued the *Guidance on Artificial Intelligence (AI) and Machine Learning (ML) technologies based Medical Devices* (“MDS-G010”).^216^

In a prior detailed analysis, the MDS-G010 was characterized as a “patchwork of existing international best practices concerning AI regulation [that] incorporates adapted forms of non-AI-based guidelines, and builds on existing legal requirements in the SFDA’s existing regulatory architecture.”^217^ The guidance has both binding and non-binding components that integrate and build on global standards from the United States FDA, the International Medical Device Regulators Forum (“IMDRF”), and the WHO, adapting them to the Saudi context.^218^ The MDS-G010 is, therefore, congruent with leading approaches, but also goes beyond them by incorporating additional best practices. Indeed, some requirements were incorporated before the FDA.^219^ Notedly, some elements of the SFDA’s architecture are binding whereas the FDA leans into soft law.^220^

The MDS-G010 is novel in several ways. It establishes requirements for manufacturers to obtain Medical Device Marketing Authorization (“MDMA”).^221^ The SFDA adopts principles related to “Software as a Medical Device” (“SaMD”) as agreed by the IMDRF, and uses those principles as a basis for evaluating AI devices.^222^ AI devices are ones that “diagnose, manage or predict diseases by analyzing medical data.”^223^ Within that scheme, the SFDA notes that there are no internationally agreed standard for clinical evaluations.^224^ To close that gap, it partially adapts standards from the WHO that other regulators have not.^225^ Several provisions are worth restating here:

The manufacturer should assess whether the promised medical benefit is achieved is consistent with the state of the art.

Manufacturers should provide assurance that metrics of effectiveness and safety include outcomes that are meaningful to patients and clinical outcome, i.e. measures of improvement in patient outcomes, clinical process or time efficiency, measures of acceptable unintended consequences, and absence of harm to patients.

The manufacturer should generate evidence on device performance that can be generalized to the entire intended population, demonstrating that performance will not deteriorate across populations and sites.

The effects of AI/ML-based medical devices should be evaluated in clinically relevant conditions, i.e. this requires integration into the existing clinical workflow

Manufacturers in their study design should consider proactively the effects that their studies may have on healthcare organizations and potentially explore the possibility of prospective real-world studies in order to minimize selection bias, have more control over variables and data collection, and examine multiple outcomes.^226^

These articles are non-binding components of the MDS-G010 because the phrases “should” and “advise” repeatedly occur.^227^ Nevertheless, the inclusion of adapted WHO guidance represents an innovation by the SFDA through the combination of hard law and soft law to close gaps where they exist. Additionally, some provisions in the MDS-G010 have a patient-centric focus despite its technical nature. The SFDA notably states that AI “may present risks that could jeopardize patient health and safety, increase inequalities and inefficiencies, undermine trust in healthcare, and adversely impact the management of healthcare.’^228^ This is a rare acknowledgement among regulators that patient trust and welfare are as crucial as technical safety.

This patient-centric emphasis is central to the TLA and should be encouraged by all regulators. Indeed, the SFDA also effectively brings the FDA’s TPLC into binding practice through its requirement that manufacturers institute “post-market continuous monitoring of safety, effectiveness, and performance” for AI devices operating in the real world.^229^ On risk management, manufacturers are also required to “demonstrate that their medical devices do not pose unacceptable risks, and that the benefits of their intended use outweigh the overall residual risk.”^230^

The FDA has to date cleared AI devices through existing pathways like 510(k) and de novo.^231^ This has led to concerns about “predicate creep” whereby new AI devices get cleared based on comparisons with older non-AI devices, which raises safety concerns.^232^ The SFDA has been more proactive and decisive by incorporating bespoke standards for AI.^233^ This approach clearly goes further than the EU because the AI Act does “not address matters of health directly relevant to AI-based medical devices.”^234^ Nevertheless, the unresolved binding nature of the MDS-G010 is noteworthy. There are binding “components” in the MDS-G010 that arise from underlying medical device law,^235^ but there are also non-binding international best practices.^236^ This hybrid approach is innovative but may blur lines of accountability, creating challenges in delineating stakeholders’ responsibilities. For example, determining whether a lapse is a legal violation or a best practice gap.

The key point to emphasize is that the SFDA’s approach is novel in certain aspects and takes on a patient-centric focus. That is important for the TLA which seeks to weave a consistent focus on individual rights. The requirements for market approvals will be necessarily more technical in nature but the language and philosophy of approach are also important. Market approval should not merely be a tick box technical exercise concerning risk but should feed into a broader narrative of patient safety. The SFDA’s approach is also congruent with the TLA because it also stipulates post-market monitoring and a focus on the continuous learning of AI systems.^237^ Embedding the SFDA’s approach within the TLA’s device approval phase represents a forward-thinking patient-centric approach. The Saudi example demonstrates that AI device approvals can integrate global best practices while being tailored to national context. Its hybrid binding structure – though imperfect – might also be an approach for low- and middle-income countries to follow, to leapfrog older models by adopting select innovations from multiple jurisdictions.

### 3.3. Post Implementation and the UAE Example

The final part of the TLA applies once AI is deployed and integrated into health care practice. Abu Dhabi^238^ and Dubai^239^ have addressed such governance through two “policies” which were among the first in the world on post implementation in health care. These exist within a much broader framework of developments for AI governance in the UAE.^240^ While termed as policies, they contain binding requirements with penalties for lack of compliance.^241^ As such, these are not voluntary best practices. The policies are similar in focus, but Dubai’s offers more depth and will therefore be primarily outlined below.

Both policies are broad in scope and contain clear requirements to protect patients and ensure accountability for the use of AI. Both policies apply to all health care providers, pharmaceutical manufacturers, insurers, researchers, and AI developers using data from local health care systems.^242^ Dubai’s policy requires that “all AI solutions for healthcare” must conform to the relevant local and international laws and regulations, “with respect to values, patient autonomy, people rights, and acceptable ethics.”^243^ AI must be free of biases, and accountability for AI outputs in health care “must be agreed between designer, researcher, developer, operators/ commissioners, and end users.”^244^ AI must also have in-built appeals processes for users to challenge significant decisions.^245^ Abu Dhabi has similar provisions that seek to “minimize any potential risks to patient safety.”^246^

There are important requirements on transparency, such as disclosing which data sets were used, what the role of the health care professional is in making the final decision, an ethical examination of how the data is used, and how the AI solution must be integrated into health care provision.^247^ The emphasis on patients is robust, with the recognition that AI solutions for health care “may directly impact people’s lives in a significant way” and so “must be designed with utmost care.”^248^ The policy imposes “minimum acceptable requirements for AI tools” that encompass compliance with a plethora of laws, namely, federal laws and regulatory requirements pertaining to telehealth, data protection, and cybersecurity, as well as, an ICT health law, a medical liability law, human research laws, health insurance law, and more.^249^ In this manner, the policy makes post-market AI governance an extension of the legal frameworks governing health care. It moves beyond other frameworks by giving regulatory oversight to the Dubai Health Authority (“DHA”).^250^ End users must report incidents, deficiencies, and issues arising from the implementation of AI to the DHA.^251^ It is also the responsibility of the DHA to create a regulatory framework that governs AI in health care, to monitor compliance with the policy through reporting, audits, and inspections, and to impose sanctions for breaches.^252^

If these policies become a model for other countries to follow, then hospitals and other stakeholders will need internal processes to ensure compliance with continuous monitoring and reporting requirements. The requirements on patient redress mechanisms or appeals procedures will require some thought to ensure consistency in application of those mechanisms across institutions to ensure proper compliance with the law. Perhaps Cohen et al.’s proposal for an appeals process (noted supra) could indicate the path forward here.^253^

Another approach could be to create an AI Bill of Rights that connects patient protections clearly and holistically to the ethical and legal obligations that surround stakeholders.^254^ Some jurisdictions have a patient bill of rights, including Qatar,^255^ Saudi Arabia,^256^ the UAE,^257^ the UK,^258^ the United States,^259^ and many other countries. These existing patient charters could be updated with an AI Bill of Rights, with provisions on informed consent, the right to an explanation for the use of AI, rights concerning data use with AI and transparency, and a straightforward right to redress for patients to pursue (such as complaining to an AI committee in a hospital that can resolve or escalate matters on behalf of patients through appropriate ethics or legal channels).^260^ Patients could be given the right to opt out of AI being used in their care where appropriate human alternatives exist. These could be formulated in a manner that is congruent with the expectations of regulators such as the authorities in Dubai and Abu Dhabi, with their focus on protecting patients.

By institutionalizing rights in this manner, the gap can be closed between high level principles and complex legal systems to the on-the-ground patient experience. A Bill of Rights supports the TLA’s requirement that law, and ethics must be central considerations from research and development through to deployment. Ultimately, the UAE’s post-implementation framework illustrates how binding patient protection measures can be embedded in national AI governance, and how such provisions could be adapted in other jurisdictions to make lifecycle approaches tangible for patients.

## 4. The Future of the TLA

While the TLA offers a pathway for governing health care AI, it is not without its limitations. Highlighting those limitations candidly is essential to refining the framework and encouraging constructive debate. As such, this paper closes by critiquing the potential shortcomings of the TLA before synthesizing its strengths to demonstrate how it exemplifies a comprehensive and interconnected holistic governance model.

One of the main concerns noted above relates to where certain phases of the TLA fit into the overall governance architecture. For example, the first phase of research and development might risk replicating IRB functions or unnecessarily overburdening them. IRBs already evaluate matters of ethics, informed consent, data privacy, and so on.^261^ Also, even if an AI committee is created, one then must deal with the obvious challenge in finding relevant experts for such committees. Many regions will not have many experts spanning AI, ethics, law, and health care to evaluate relevant systems.^262^ Even in well-resourced Gulf countries, finding such talent is difficult, but in low resource settings, creating more governance could exacerbate inequities.^263^ This risk underscores the need for proportionate implementation. The TLA’s phases can be embedded into existing review or audit processes rather than creating standalone bureaucracies, allowing for context-sensitive adoption.

The examples of Qatar, Saudi Arabia, and the UAE work, in part, because they have compact health care ecosystems and there can be strong centralized government coordination (although, that does not always translate to greater efficiency). In larger dynamic economies like the United States and the EU, there are more diverse stakeholders, siloed oversight, and rapid innovation cycles. For example, the first phase of the TLA on research and development would be difficult to integrate into the FDA’s TPLC because of existing layers of bureaucracy. The EU’s AI Act has already introduced heavy regulatory demands on stakeholders^264^ – injecting TLA governance could further increase costs and complexity. Proportionality also means recognizing when certain elements can be scaled back or merged with existing requirements. For instance, in jurisdictions already operating robust post-market monitoring, the TLA’s post-implementation checks could be incorporated as targeted enhancements rather than wholesale additions.

Along those lines, it may also simply be unnecessary to have TLA governance, creating more red tape and overcomplicated governance. There are already lifecycle frameworks noted above that emphasize post market monitoring.^265^ Implementing the TLA could lead to fragmented oversight over the different phases and may burden manufacturers and those subject to regulations, duplicating documentation without enhancing patient safety. It could be argued that the SFDA’s MDS G010 already incorporates adapted forms of best practices from the WHO and others. Ultimately, proportionate integration and adaptive implementation are key to ensuring that the TLA does not duplicate or overburden but instead strengthens patient protections without stifling innovation.

Despite these limitations, the TLA can move beyond them. This framework should cause us to think about how we can create a coherent and unified approach that puts patients first and closes the gaps that exist in the existing piecemeal approaches. AI brings with it specific challenges in health care and patients should not be ignored at any stage of the AI lifecycle. Some frameworks ignore them,^266^ while others only address their concerns in passing.^267^ In an earlier paper, it was noted how the examples from Qatar, Saudi Arabia, and the UAE exemplify the “interconnectedness of the [TLA].”^268^ By creating a patient-centered approach that emphasizes law and ethics “each regulatory phase reinforces and builds upon the others.”^269^

Thus, compliance with Qatar’s guidelines during research and development with its rigorous documentation on matters such as data biases, and compliance with laws such as those on data protection, would prepare developers for obtaining market approval later. For example, the SFDA requires that AI devices work on their intended population, that biases are mitigated, and that performance is evaluated in clinical practice.^270^ Obtaining certification through Qatar-style guidelines with its record keeping on ethical justifications for the AI system and data limitations, could provide the evidence required for SFDA risk assessments without the need for duplication, and may reduce the risk of devices being rejected given that best practices would have been followed. The emphasis on transparency in the Qatar guidelines would also support the post-market monitoring requirements under the SFDA architecture. Addressing biases early would avoid making costly adjustments later when an SFDA audit reveals concerns.

The same is true for post-implementation requirements that move beyond mere market approval of devices. The UAE example highlights how more governance can be expected beyond FDA-style regulators, with regulators potentially having far broader powers to monitor the use of AI in practice. For example, the UAE policies require that AI is free from biases, transparent, and compliant with medical liability laws.^271^ An AI system that has navigated Qatar-style guidelines at the research stage and SFDA style regulations on device approval will be prepared for downstream regulatory enquiries. If documentation on explainability is in place, and there are accountability mechanisms built in to facilitate an appeals process, then this would support the requirements of the DHA. Consider an AI system used in a hospital that hallucinates, makes an incorrect recommendation, and ultimately harms the patient. There will be foundational R&D records in place to ensure efficient reporting to the DHA which would assist with determining corrective actions. In this manner, rigorous ethical R&D at the outset would help streamline approvals and support post-market trust and redress. This interconnectedness can be seen in Table 2.

**Table 2.** Interconnectedness of TLA Phases

Phase	Key Elements	How it Facilitates the Next Phase
R&D (Qatar Guidelines)	Ethical rigor, bias documentation, data protection compliance	Provides evidence for SFDA risk assessments, reducing rejections.
Approval (SFDA)	Patient-centric metrics, post-market monitoring	Ensures bias-free, transparent devices for UAE deployment.
Post-Implementation (UAE)	Appeals, reporting to DHA	Builds on prior records for swift harm resolution, enhancing trust.

At this stage then, the TLA may be thought of as a dynamic and evolving framework that weaves patient centric principles through its three phases from the lab to clinic. By focusing on patients in this systematic way, it goes beyond what FDA and EU frameworks can do in health care. Actionable rights are fundamental considerations throughout, such as opt outs and appeals, and this complements the WHO guidance by encouraging practical tools that apply at the national level. By synthesizing these pioneering approaches, the TLA offers a template that can be adapted globally to protect patients wherever AI is used in health care. Through Qatar’s approach to research and development, Saudi Arabia’s forward-thinking medical device approvals frameworks, and the UAE’s comprehensive obligations post-deployment, the TLA offers a model to remedy existing gaps in governance that focus on protecting patients. The TLA is not perfect, but the aim is to stir debate about health care AI governance, urge global collaboration, inspire similar national-level initiatives, and demonstrate that models trialed in the Gulf can inform AI governance in health care worldwide.

## Footnotes

1 Barry Solaiman et al., *A “True Lifecycle Approach” Towards Governing Healthcare AI with the GCC as a Global Governance Model*, 8 npj Digit. Med., June 5, 2025, at 1, 1 https://www.nature.com/articles/s41746-025-01614-1 [https://doi.org/10.1038/s41746-025-01614-1] [hereinafter *A True Lifecycle Approach*].

2
*Id.*

3
*See* Sara Gerke, Timo Minssen & I. Glenn Cohen, *Ethical and Legal Challenges of Artificial Intelligence-Driven Healthcare*, *in* Artificial Intelligence in Healthcare 295, 328 (Adam Bohr & Kaveh Memarzadeh eds., 2020), https://www.sciencedirect.com/science/article/pii/B9780128184387000125?via%3Dihub [https://doi.org/10.1016/B978-0-12-818438-7.00012-5] (describing components needed to create effective regulations around AI-driven health care).

4 Martin Ebers, *Standardizing AI - The Case of the European Commission’s Proposal for an Artificial Intelligence Act*, *in* The Cambridge Handbook of Artificial Intelligence Global Perspectives on Law and Ethics 321, 323-25 (Larry A. DiMatteo, Cristina Poncibò & Michel Cannarsa eds., 2022).

5
*See* Barry Solaiman & I. Glenn Cohen, *A Framework for Health, AI and the Law* (discussing study evaluating AI in health settings regarding use case and accuracy), *in* Research Handbook on Health, AI and the Law 1, 3 (Barry Solaiman & I. Glenn Cohen eds., 2024), https://doi.org/10.4337/9781802205657.ch01.

6
*See* Brian Anderson & Eric Sutherland, *Collective Action for Responsible AI in Health* 8-9 (Org. for Econ. Coop. and Dev., OECD Artificial Intelligence Papers No. 10, 2024), https://www.oecd.org/content/dam/oecd/en/publications/reports/2024/01/collective-action-for-responsible-ai-in-health_9a65136f/f2050177-en.pdf [https://perma.cc/CR7P-AM6P] (explaining how AI is improving data synthesis, increasing efficiency, and more in health care).

7 Paige Nong et al., *Current Use and Evaluation of Artificial Intelligence and Predictive Models in U.S. Hospitals*, 44 Health Affs. 90, 90 (2025).

8
*Artificial Intelligence*, NHS Eng.: Transformation Directorate (Apr. 30, 2025), https://transform.england.nhs.uk/information-governance/guidance/artificial-intelligence/ [https://perma.cc/94TX-EKJ8].

9 Excellence First Enter. Consultancy, *China’s AI Healthcare Revolution: What UK Partners Should Know*, Cambridge Network (June 9, 2025), https://www.cambridgenetwork.co.uk/news/chinas-ai-healthcare-revolution-what-uk-partners-should-know [https://perma.cc/Y8Q7-H6SZ].

10 Asif Dhar, Bill Fera & Leslie Korenda, *Can GenAI Help Make Health Care Affordable? Consumers Think So*, Health Forward Blog (Nov. 16, 2023), https://www.deloitte.com/us/en/Industries/life-sciences-health-care/blogs/health-care/can-gen-ai-help-make-health-care-affordable-consumers-think-so.html [https://perma.cc/Z85Q-P594].

11
*Artificial Intelligence*, *supra* note 8.

12
*Id.*; *Medical Devices & Digital Tools*, NHS Eng. (June 2, 2025), https://www.england.nhs.uk/long-read/medical-devices-and-digital-tools/ [https://perma.cc/S7EP-4R8R].

13
*NHS Artificial Intelligence (AI) Giving Patients Better Care and Support*, NHS Eng.: News (Dec. 12, 2024), https://www.england.nhs.uk/2024/12/nhs-ai-giving-patients-better-care-and-support/ [https://perma.cc/B9U8-WCT9].

14
*See* Anderson & Sutherland, *supra* note 6, at 21-23 (describing the various opportunities for AI in health care once risk controls are made effective).

15
*A True Lifecycle Approach*, *supra* note 1.

16
*See* Barry Solaiman, *From ‘AI to Law’ in Healthcare: The Proliferation of Global Guidelines in a Void of Legal Uncertainty*, 42 Med. & L. 391, 399-400 (2023) (demonstrating that AI guidelines and policies are becoming more widespread across the world).

17
*See id.* at 5-7 (highlighting legal scholars research on informed consent, disclosure, and medical liability).

18
*See generally* World Health Org., Ethics and Governance of Artificial Intelligence for Health: WHO Guidance (2021) (WHO guidelines to ensure AI works for public benefit), https://iris.who.int/server/api/core/bitstreams/f780d926-4ae3-42ce-a6d6-e898a5562621/content [https://perma.cc/6DYD-TDV5]; Org. for Econ. Coop. & Dev., AI in Health: Huge Potential, Huge Risks (2024), https://www.oecd.org/content/dam/oecd/en/publications/reports/2024/01/ai-in-health-huge-potential-huge-risks_ff823a24/2f709270-en.pdf [https://perma.cc/RZD9-ZQ4P] (highlighting OECD nations defining policy principles for use in AI since 2019); *AI Principles*, OECD, https://www.oecd.org/en/topics/sub-issues/ai-principles.html (last visited Aug. 13, 2025).

19 Regulation (EU) 2024/1689 of June 13, 2024, Laying Down Harmonised Rules on Artificial Intelligence (Artificial Intelligence Act), 2024 O.J. (L) 1 [hereinafter AI Act]; Directive 2024/2853 of Oct. 23, 2024, On Liability for Defective Products, 2024 O.J. (L) 1.

20
*See* Antoni Lorente, *Institutional Review Boards as Soft Governance Mechanisms of R&D: Governing the R&D of AI-based Medical Products*, 1 J. AI L. & Reg. 40, 40 (2024), https://arxiv.org/pdf/2410.19574 [https://doi.org/10.48550/arXiv.2410.19574] (discussing how risk based approaches to governing AI are unclear especially regarding clear risks in final products).

21 Abeer Malik & Barry Solaiman, *AI in Hospital Administration and Management: Ethical and Legal Implications*, *in* Research Handbook on Health, AI and the Law, *supra* note 5, at 21, 31.

22
*See* Brian J. Wells et al., *A Practical Framework for Appropriate Implementation and Review of Artificial Intelligence (FAIR-AI) in Healthcare*, 8 npj Digit. Med. Aug. 11, 2025, at 1, 1, https://www.nature.com/articles/s41746-025-01900-y [https://doi.org/10.1038/s41746-025-01900-y] (describing how health systems in both the US and EU do not have current AI governance models).

23
*See* Pers. Data Prot. Comm’n, Compendium of Use Cases: Practical Illustrations of the Model AI Governance Framework 30-31 https://www.pdpc.gov.sg/-/media/files/pdpc/pdf-files/resource-for-organisation/ai/sgaigovusecases.pdf [https://perma.cc/PQ2L-E9KS] (Sing.) (showing an example of a start-up UCARE.AI developing accountable AI for use in health care with strong governance).

24 Wells et al., *supra* note 22, at 1.

25 Wells et al., *supra* note 22, at 1.

26
*A True Lifecycle Approach, supra* note 1.

27
*Id.*

28
*See generally* Barry Solaiman et al., *Regulating AI in Health in the Middle East: Case Studies from Qatar, Saudi Arabia and the United Arab Emirates* (investigating countries like Qatar, UAE, and Saudi Arabia to create an empirical analysis of their advancing AI governance), *in* Research Handbook on Health, AI and the Law, *supra* note 5, at 332, 332-54.

29
*A True Lifecycle Approach, supra* note 1.

30
*See* Exec. Order No. 13,859, 84 Fed. Reg. 3697 (Feb. 14, 2019) (advocating a free-market approach to AI development). *See also* Exec. Order No. 14,179, 90 Fed. Reg. 8741 (Jan. 23, 2025) (revoking Biden‑era AI regulations and mandating an AI Action Plan).

31
*See* Barry Solaiman, *The European Union’s Artificial Intelligence Act and Trust: Towards an AI Bill of Rights in Healthcare?*, 17 L. Innov. & Tech. 318, 318-19 (2025), https://www.tandfonline.com/doi/full/10.1080/17579961.2025.2469986 [https://doi.org/10.1080/17579961.2025.2469986] [hereinafter *The EU’s AI Act and Trust*] (discussing how the AI-Act through revisions and additions creates unnecessary complexity).

32
*See* Huw Roberts et al., *The Chinese Approach to Artificial Intelligence: An Analysis of Policy, Ethics, and Regulation*, 36 AI & Soc’y 59, 60 (2021), https://link.springer.com/article/10.1007/s00146-020-00992-2 [https://doi.org/10.1007/s00146-020-00992-2] (analyzing China’s Artificial Intelligence Development Plan (AIDP) and its unified policy objectives with goal to lead AI race); Mimi Zou & Lu Zhang, *Navigating China’s Regulatory Approach to Generative Artificial Intelligence and Large Language Models*, 1 Cambridge F. on AI: L. & Governance, May 16, 2025, at 1, 2, https://www.cambridge.org/core/journals/cambridge-forum-on-ai-law-and-governance/article/navigating-chinas-regulatory-approach-to-generative-artificial-intelligence-and-large-language-models/969B2055997BF42DE693B7A1A1B4E8BA [https://doi.org/10.1017/cfl.2024.4] (describing China’s overarching state led goals for AI in relation to social, economic, and industrial goals).

33
*See* Tom L. Beauchamp & James F. Childress, Principles of Biomedical Ethics 118-19 (8th ed. 2019) (explaining the justifications for informed consent and patient autonomy).

34
*See* Barry Solaiman & Abeer Malik, *Regulating Algorithmic Care in the European Union: Evolving Doctor–Patient Models Through the Artificial Intelligence Act (AI-Act) and the Liability Directives*, 33 Med. L. Rev. 1, 1 (2025), https://academic.oup.com/medlaw/article/33/1/fwae033/7754853 [https://doi.org/10.1093/medlaw/fwae033] (discussing how western medicine emphasizes patient autonomy in a consumeristic sense); Montgomery v. Lanarkshire Health Bd. [2015] UKSC 11, [81] (UK) (finding patients can accept and understand risk related to medical care moving away from medical paternalism).

35 Barry Solaiman, *Assessing Healthcare Rights and Responsibilities Under the Constitutional Orders of Mainland China and the Special Administrative Regions*, *in* Routledge Handbook of Constitutional Law in Greater China 266, 271-72 (Ngoc Sun Bui, Stuart Hargreaves & Ryan Mitchell eds., 2023). *See also* Barry Solaiman & Suhaila Ghuloum, *Towards Community Care: Qatar’s Rapidly Evolving Mental Health Landscape*, 19 BJPsych Int’l 15, 16 (2022) (UK) (noting that the laws of Qatar have to balance “patient autonomy with family interventions and safety for the patient, society and healthcare providers”).

36 Beauchamp & Childress, *supra* note 33, at 158-59, 218-19. *See also* World Med. Org., Declaration of Geneva 2 (2017) (stating that the doctor shall respect the patient and their life as well as not use their expertise to harm the rights and liberty of others).

37
*See Ancient Greek Medicine “I Swear by Apollo Physician…”: Greek Medicine from the Gods to Galen*, NIH Nat’l Libr. of Med., https://www.nlm.nih.gov/hmd/topics/greek-medicine/index.html#case1 [https://perma.cc/5DBC-42HG] (last visited Sep. 26, 2025) (noting that the Hippocratic Oath creates certain professional standards, including one to benefit patients and to not do any harm to them); World Med. Org., *supra* note 36 (highlighting principles of acting in the interest of patients and promising to do no harm to them); B. Sonny Bal, *An Introduction to Medical Malpractice in the United States*, 467 Clin. Orthop. & Related Rsch. 339, 339, 342 (2008) (stating the elements of a medical malpractice claim, with one in particular being the duty owed to the patient, as well as discussing the history of medical malpractice and how it was derived from the higher level of professional responsibility that doctors have).

38
*See* Tom Vandersteegen, Wim Marneffe, Irina Cleemput & Lode Vereeck, *The Impact of No-Fault Compensation on Health Care Expenditures: An Empirical Study of OECD Countries*, 119 Health Pol’y 367, 368, 370 (2015) (stating that New Zealand and certain Nordic countries have no-fault liability schemes and that there are only eight countries with a no-fault liability system).

39 Regulation (EU) 2016/679 of April 27, 2016, On the Protection of Natural Persons with Regard to the Processing of Personal Data and on the Free Movement of Such Data (General Data Protection Regulation), art. 9, 2016 O.J. (L 119) 38; *What is Special Category Data?*, Info. Comm’r’s Off. (Apr. 9, 2024), https://ico.org.uk/for-organisations/uk-gdpr-guidance-and-resources/lawful-basis/special-category-data/what-is-special-category-data/ [https://perma.cc/RVN6-ZKJS]. *See also Summary of the HIPAA Privacy Rule*, U.S. Dep’t of Health & Hum. Servs. (Mar. 14, 2025), https://www.hhs.gov/hipaa/for-professionals/privacy/laws-regulations/index.html [https://perma.cc/E84S-RXQG] (stating that the HIPPA Privacy Rule is intended to balance the privacy of health information while also allowing it to be transferred in certain circumstances to protect public health, which is why it needs a unique set of rules).

40 Regulation (EU) 2016/679, *supra* note 39, at 38.

41 Ashworth Sec. Hosp. v M.G.N. Ltd. [2000] 1 WLR 515, [47] (Lord Philips MR) (appeal taken from QB) (UK).

42 Zhonghua Renming Jiben Yiliaoweisheng Yu Jiankang Cujin Fa (中华人民共和国基本医疗卫生与健康促进法) [Law on Basic Medical and Health Care and Promotion of Health] (Promulgated by the Standing Comm. of the Nat’l People’s Cong., Dec. 28, 2019, effective June 1, 2020), art. 33, 2020 Standing Comm. Nat’l People’s Cong. Gaz 46 (China).

43 International Covenant on Economic, Social and Cultural Rights art. 12, Dec. 16, 1966, 993 U.N.T.S. 8.

44
*Id.* art. 2.

45
*See id.* (noting the importance of equality in the right to health by prohibiting certain types of discrimination); United Nations Educational, Scientific and Cultural Organization [UNESCO] 33 C/Resolution 36, at art. 10, Universal Declaration on Bioethics and Human Rights (Oct. 19, 2005) (stating that all people should be treated with equal dignity and rights to ensure justice and equity).

46
*Id.*

47
*Id.* at art. 3.

48
*See, e.g.*, 21 C.F.R. Pts. 800-98 (2025) (medical device regulations); Medical Device Act, amended by Act. No. 15279, Dec. 19, 2017 (S. Kor.), *translated in* Ministry of Food and Drug Safety’s online website, https://www.mfds.go.kr/eng/brd/m_40/list.do [https://perma.cc/H5CX-JEJR] (search required); Saudi Food and Drug Auth., *Implementing Regulation of the Law of Medical Devices*, SFDA Board Decision No. 3-29-1443 (Sep. 26, 2021).

49
*See generally* World Health Org., WHO Global Benchmarking Tool + Medical Devices (GBT + Medical Devices) for Evaluation of National Regulatory Systems of Medical Devices and In-Vitro Diagnostics 6-9, 34-47 (2024) (increasing extent of regulations are an indicator of higher maturity levels in regard to medical devices); World Health Org., List of National Regulatory Authorities (NRAs) Operating at Maturity Level 3 (ML3) and Maturity Level 4 (ML4) (as Benchmarked Against WHO Global Benchmarking Tool (GBT)) (2025), https://cdn.who.int/media/docs/default-source/medicines/regulatory-systems/wla/list-of-nras-operating-at-ml3-and-ml4.pdf [https://perma.cc/8F7M-GDDJ] (including only eighteen countries that have either a maturity level of 3 or 4 regarding medical device regulations).

50 Solaiman & Cohen, *supra* note 5, at 3.

51
*See* Boris Babic, Sara Gerke, Theodoros Evgeniou & I. Glenn Cohen, *Algorithms on Regulatory Lockdown in Medicine*, 336 Sci. 1202, 1202 (2019) (noting that adaptive AI systems learn and change over time, which can allow for performance calibrations in the AI systems).

52
*See id.* at 1203 (noting that the FDA has only approved “locked” algorithms for medical devices). *See also* Meghavi Mashar et al., *Artificial Intelligence Algorithms in Health Care: Is the Current Food and Drug Administration Regulation Sufficient?*, 2 JMIR AI, 2023, at 1, 1 (stating that FDA approved devices with machine learning only have locked algorithms).

53 Solaiman & Cohen, *supra* note 5, at 3.

54
*See* Babic, Gerke, Evgeniou & Cohen, *supra* note 51, at 1202-03 (referencing how adaptive AI algorithms can unintentionally be changed when faulty data is added to it, for example, this could occur if a physician found a way to guarantee a “low risk of overdose” score when trying to get their patient a prescription opioid).

55
*See* W. Nicholson Price II, Sara Gerke & I. Glenn Cohen, *Liability for Use of Artificial Intelligence in Medicine* (noting that AI could make better recommendations than the standard of care, potentially shifting the standard, but whether this happens will likely depend on the practice area and specific use), *in* Research Handbook on Health, AI and the Law, *supra* note 5, at 150, 153-54; Malik & Solaiman, *supra* note 21, at 36 (stating that the importance of patient and staff data for administrative purposes is critical and that the collection of that data for AI use could lead to additional risk of a data breach).

56
*See* Solaiman & Cohen, *supra* note 5, at 3 (discussing that explainability creates a problem in the AI landscape because certain AIs have a “black box” model that cannot be interpreted); Boris Babic, Sara Gerke, Theodoros Evgeniou & I. Glenn Cohen, *Beware Explanations from AI in Health Care*, 373 Sci. 284, 284 (2021) (noting that black-box AI models that cannot be interpreted are a cause of skepticism and distrust among many users).

57
*Informed Consent*, Am. Cancer Soc’y (July 25, 2025), https://www.cancer.org/cancer/managing-cancer/making-treatment-decisions/informed-consent.html [https://perma.cc/Y5LQ-RU2L].

58
*See id.* (stating that providers may use different approaches to explain a patient’s treatment options, that patients should ask for explanations of confusing terms, and that patients should request information in their preferred language).

59
*See generally* Barry Solaiman & Mark G. Bloom, *AI, Explainability, and Safeguarding Patient Safety in Europe: Toward a Science-Focused Regulatory Model*, *in* The Future of Medical Device Regulation: Innovation and Protection (I. Glenn Cohen et al. eds., 2022) (describing that AI explainability means the ability for people to understand and explain an AI’s rationale for its decisions).

60 Rebeca Tenajas & David Miraut, *The Hidden Risk of AI Hallucinations in Medical Practice*, Annals of Fam. Med. (Mar. 16, 2025), https://www.annfammed.org/content/hidden-risk-ai-hallucinations-medical-practice [https://perma.cc/2VQ2-V3D2]; Christine J. Colasacco & Hayley L. Born, *A Case of Artificial Intelligence Chatbot Hallucination*, 150 JAMA Otolaryngol. Head Neck Surg. 457, 457 (2024), https://jamanetwork.com/journals/jamaotolaryngology/article-abstract/2817762 [https://doi.org/10.1001/jamaoto.2024.0428]; Rami Hatem, Brianna Simmons & Joseph E. Thornton, *A Call to Address AI “Hallucinations” and How Healthcare Professionals Can Mitigate Their Risks*, 15 Cureus, Sep. 5, 2023, at 1, 1 https://www.cureus.com/articles/183657-a-call-to-address-ai-hallucinations-and-how-healthcare-professionals-can-mitigate-their-risks#!/ [https://doi.org/10.7759/cureus.44720].

61
*See* Barry Solaiman, *Generative Artificial Intelligence (GenAI) and Decision-Making: Legal & Ethical Hurdles for Implementation in Mental Health*, 97 Int’l J.L. & Psychiatry 1, 4 (2024), https://www.sciencedirect.com/science/article/pii/S0160252724000773?via%3Dihub [https://doi.org/10.1016/j.ijlp.2024.102028] (explaining that AI has been shown to make dangerous recommendations given it can sometimes be misleading or inaccurate, which can potentially lead to suicide, and at its most extreme AI can hallucinate, meaning it makes up information, which could end up being used by a clinician); Ismail Dergaa et al., *ChatGPT Is Not Ready Yet for Use in Providing Mental Health Assessment and Interventions*, 14 Frontiers Psychiatry 1, 4 (2024) (noting that in scenario B, ChatGPT failed to ask clarifying questions regarding the patient’s symptoms and did not consider or rule out depression, which could potentially increase the risk of suicide).

62
*See* Montgomery v. Lanarkshire Health Bd. [2015] UKSC 11, [82-85], [2015] 2 WLR 768 (appeal taken from Scotland) (UK) (discussing the law of negligence in the United Kingdom and how doctors have a duty to take reasonable care to ensure that a patient is aware of certain risks involved in medical treatments).

63
*See* Zahra Sadeghi et al., *A Review of Explainable Artificial Intelligence in Healthcare*, 118 Computs. & Elec. Eng’g, Aug. 2024, at 1, 13 (listing the different challenges regarding AI explainability, with those challenges going beyond expertise in mathematics and statistics, including how transparency lessens efficiency and how being able to identify relevant parts of the AI still leaves the question of why those parts are relevant).

64
*See, e.g.*, Corrigan v. Covidien L.P., 748 F. Supp. 3d 1, 12 (D. Mass. 2024) (stating that the learned intermediary rule requires a medical device manufacturer to warn a physician instead of the patient directly).

65 W. Nicholson Price II, *Artificial Intelligence in Health Care: Applications and Legal Implications*, 14 SciTech Law. 10, 13 (2017).

66
*See* Solaiman & Cohen, *supra* note 5, at 4 (stating that decentralized systems are less vulnerable to adversarial attacks since they have data in different locations, while centralised systems do not and only have one point of entry). *See also* Barry Solaiman & Georgios Dimitropoulos, *The Legal Considerations of AI-Blockchain for Securing Health Data* (noting that blockchain is used for recordkeeping and that there are two main types, public and private, with the public blockchain systems being more accessible since they are not managed by any entity while private systems are more insulated since they are managed by either one or more companies), *in* Research Handbook on Health, AI and the Law, *supra* note 5, at 130, 134.

67
*See* Solaiman & Cohen, *supra* note 5, at 4 (stating that centralized systems are more vulnerable to adversarial attacks since compared to a decentralized system which has data in different places, the data in a centralized system is stored in one location); Elisabetta Biasin, Erik Kamenjašević & Kaspar Rosager Ludvigsen, *Cybersecurity of AI Medical Devices: Risks, Legislation, and Challenges* (noting that as medical devices become more integrated with the internet, cybersecurity will have to be improved to protect the health of patients), *in* Research Handbook on Health, AI and the Law, *supra* note 5, at 57, 58.

68 Solaiman & Dimitropoulos*, supra* note 66.

69
*Id.*

70 Mirja Mittermaier, Marium M. Raza & Joseph C. Kvedar, *Bias in AI-based Models for Medical Applications: Challenges and Mitigation Strategies*, 6 npj Digit. Med., June 14, 2023, at 1, 1, https://www.nature.com/articles/s41746-023-00858-z [https://doi.org/10.1038/s41746-023-00858-z].

71 See, Jessica L. Roberts & Peter Salib, *Algorithmic Discrimination and Health Equity*, *in* Research Handbook on Health, AI and the Law, *supra* note 5, at 92-110.

72 Case C-203/22, CK v. Magistrat der Stadt Wien, ECLI:EU:C:2025:142, ¶ 60 (Feb. 27, 2025).

73
*Id.* ¶¶ 68-69.

74 Solaiman & Cohen, *supra* note 5, at 3. *See generally* Krzysztof Budzyń et al., *Endoscopist Deskilling Risk After Exposure to Artificial Intelligence in Colonoscopy: A Multicentre, Observational Study*, 10 Lancet Gastroenterol. Hepatol. 896 (2025), https://www.thelancet.com/journals/langas/article/PIIS2468-1253(25)00133-5/fulltext [https://doi.org/10.1016/S2468-1253(25)00133-5] (discussing overreliance leading to deskilling).

75 Gerke, *supra* note 3, at 295.

76
*Id.*

77
*Id.*

78 Solaiman & Cohen, *supra* note 5.

79
*Id.* at 269.

80 World Health Org., Regulatory Considerations on Artificial Intelligence for Health (2023), https://iris.who.int/bitstream/handle/10665/373421/9789240078871-eng.pdf?sequence=1 [https://perma.cc/6KLE-MU7V].

81
*Id.* at 48-49.

82
*Id.* at xii (alteration in original).

83
*Id.* at 13.

84
*Id.* at 12.

85
*Total Product Life Cycle for Medical Devices*, U.S. Food & Drug Admin. (Sep. 6, 2023), https://www.fda.gov/about-fda/cdrh-transparency/total-product-life-cycle-medical-devices.

86
*Id.*

87 U.S. Food & Drug Admin., Artificial Intelligence-Enabled Device Software Functions: Lifecycle Management and Marketing Submission Recommendations: Draft Guidance for Industry and Food and Drug Administration Staff (2025) [hereinafter A.I. Enabled Device Software Functions Draft Guidance].

88
*Id.* at 3.

89
*Id.* at 5.

90 U.S. Food & Drug Admin., Health Can. & Meds. & Healthcare Prods. Regul. Agency, Good Machine Learning Practice for Medical Device Development: Guiding Principles (2021) [hereinafter GMLP].

91
*Id.* at 2.

92 A.I. Enabled Device Software Functions Draft Guidance, *supra* note 87, at 5.

93 GMLP, *supra* note 90, at 2.

94 A.I. Enabled Device Software Functions Draft Guidance, *supra* note 87, at 18.

95
*Id.* at 19.

96 U.S. Food & Drug Admin., Health Can. & Meds. & Healthcare Prods. Regul. Agency, Transparency for Machine Learning-Enabled Medical Devices: Guiding Principles (2024).

97
*Id.* at 2.

98
*Id.*

99
*Id.* at 1.

100
*Id.* at 2.

101 U.S. Food & Drug Admin., Marketing Submission Recommendations for a Predetermined Change Control Plan for Artificial Intelligence-Enabled Device Software Functions: Guidance for Industry and Food and Drug Administration Staff (2024).

102
*Id.* at 11.

103 U.S. Food & Drug Admin., Health Can. & Meds. & Healthcare Prods. Regul. Agency, Predetermined Change Control Plans for Machine Learning-Enabled Medical Devices: Guiding Principles 1-2 (2023).

104
*See* GMLP, *supra* note 90, at 2.

105
*Id.* at 2.

106
*Id.*

107
*Id.*

108
*Id.*

109
*Id.*

110
*Id.*

111
*See id.*

112 A.I. Enabled Device Software Functions Draft Guidance, *supra* note 87.

113
*Id.* at 32.

114
*Id.*

115
*Id.* at 33.

116
*Id.* at 32.

117
*See id.* at 7, 32-33 (emphasizing the importance of ongoing performance monitoring for AI, including within a postmarket setting, which aligns with the TPLC’s shared focus on postmarket management).

118
*See* Boris Babic et al., *A General Framework for Governing Marketed AI/ML Medical Devices*, 8 npj Digit. Med., May 31, 2025, at 1, 1, https://www.nature.com/articles/s41746-025-01717-9 [https://doi.org/10.1038/s41746-025-01717-9]; *Manufacturer and User Facility Device Experience (MAUDE) Database*, U.S. Food & Drug Administration, https://www.accessdata.fda.gov/scripts/cdrh/cfdocs/cfmaude/search.cfm (last visited Sep. 28, 2025).

119
*See* Boris Babic et al., *supra* note 118, at 1-2 (stating that the MDR system requires manufacturers to report adverse events, including device malfunctions).

120
*Id.* at 1.

121
*See id.* at 3.

122
*Id.*

123
*Id.* at 3-5.

124
*Total Product Life Cycle for Medical Devices*, U.S. Food & Drug Admin., https://www.fda.gov/about-fda/cdrh-transparency/total-product-life-cycle-medical-devices (last visited Sep. 28, 2025).

125
*See* AI Act, *supra* note 19, at art. 113; *Guidances*, U.S. Food & Drug Admin., https://www.fda.gov/regulatory-information/search-fda-guidance-documents/guidances (last visited Sep. 28, 2025).

126
*See* AI Act, *supra* note 19, ¶¶ 1, 3, 7.

127
*Id.* ¶ 1 (alteration in original).

128
*See id.* ¶¶ 1, 26, 31.

129
*See id.* ¶ 53 (stating that certain AI systems, such as those “intended to perform a narrow procedural task,” do not significantly risk harm); Hannah van Kolfschooten & Janneke van Oirschot, *The EU Artificial Intelligence Act (2024)*, 149 Health Pol’y, Nov. 2024, at 1, 2.

130
*See id.* at art. 8.

131 Regulation 2017/745 of Apr. 5, 2017, On Medical Devices, 2017 O.J. (L 117) 1 [hereinafter MDR Regulation].

132 Regulation 2017/746 of Apr. 5, 2017, On in vitro Diagnostic Medical Devices, 2017 O.J. (L 117) 176 [hereinafter IVDR Regulation].

133 AI Act, *supra* note 19, at Annex 1 (11-12).

134 MDR Regulation, *supra* note 131, ¶ 22; IVDR Regulation, *supra* note 132, ¶ 20; AI Act, *supra* note 19, at art. 2 1(e).

135 AI Act, *supra* note 19, at art. 3(20).

136
*See id.* at 50, art. 3(21), art. 3(24), art. 48.

137 MDR Regulation, *supra* note 131, ¶ 4; IVDR Regulation, *supra* note 132, ¶ 4; Nathalie A. Smuha et al., How the EU Can Achieve Legally Trustworthy AI: A Response to the European Commission’s Proposal for an Artificial Intelligence Act 39 (Aug. 5, 2021) (working paper), https://papers.ssrn.com/sol3/papers.cfm?abstract_id=3899991 [https://doi.org/10.2139/ssrn.3899991] [hereinafter *Response to European Commission*].

138
*Response to European Commission, supra* note 137, at 39*. See also* Timo Minssen et al., *Governing AI in the European Union: Emerging Infrastructures and Regulatory Ecosystems in Health* (emphasizing preexisting critiques of self-certification, questioning the system’s reliability in ensuring CE mark requirements are met for risky AI systems), *in* Research Handbook on Health, AI & the Law, *supra* note 5, at 317.

139 AI Act, *supra* note 19, at 65.

140
*Id.* at art. 3(25).

141
*Id.* at art. 72(2).

142
*Id.* at art. 10(f).

143
*Id.* at art. 10(g).

144 A.I. Bd. & Med. Device Coordination Grp., MDCG Doc. 2025-6, AIB Doc. 2025-1, Interplay Between the Medical Devices Regulation (MDR) & In Vitro Diagnostic Medical Devices Regulation (IVDR) and the Artificial Intelligence Act 2 (2025), https://health.ec.europa.eu/document/download/b78a17d7-e3cd-4943-851d-e02a2f22bbb4_en?filename=mdcg_2025-6_en.pdf [https://perma.cc/U82M-7UBV].

145
*See id.* (describing the simultaneous yet complementary application of both MDR/IVRR and AIA for medical devices with one or more high-risk AI system).

146
*See id.* at 3 (describing how manufacturers can integrate testing and reporting processes for MDAI into already established procedures under the MDR/IVDR to ensure consistency, avoid duplication and minimize added burden).

147
*See A True Lifecycle Approach, supra* note 1, at 2, 5 (discussing how, despite lifecycle approaches, EU’s AI Act and FDA’s TPLC “focus primarily on compliance without integrating standards of medical law and ethics to protect patients”).

148
*See* Solaiman and Malik, *supra* note 34, at 10 (noting “[t]he AI-Act has ‘severe weaknesses’ … .”); Timo Minssen et al., *supra* note 138, at 317 (pointing out that the “AI Act may be built upon flawed foundations, merely replicating the limitations of existing governance mechanisms”).

149 Solaiman & Malik, *supra* note 34, at 2, 10.

150
*See id.* at 15 (noting that, despite patient’s abilities to notify market surveillance authorities where AI systems post risk, there is no indication for what degree of risk necessitates a report and if no report is provide and “the patient is harmed, the provider presumably will not be responsible”).

151 Martin Ebers, *AI Robotics in Healthcare Between the EU Medical Device Regulation and the Artificial Intelligence Act: Gaps and Inconsistencies in the Protection of Patients and Care Recipients*, 11 Oslo L. Rev., Oct. 31, 2024, at 1, 11, https://www.scup.com/doi/10.18261/olr.11.1.2 [https://doi.org/10.18261/olr.11.1.2] (Nor.).

152
*The EU’s AI Act and Trust*, *supra* note 31, at 328.

153 Ebers, *supra* note 151, at 1.

154
*See id.* at 3-4 (highlighting that with regard to lifestyle and wellness devices, which are not medical devices under the MDR, “it seems problematic that no other special EU regulation exists to protect users”).

155
*Id.* at 4.

156
*Id.* at 11.

157
*Id.*

158
*See generally* Ann-Kathrin Carl & David Hochmann, *Impact of the New European Medical Device Regulation: A Two-Year Comparison*, 69 Biomed. Eng’g 317, 319, 321, 324-25 (2023) (describing challenges including increased resource expenditure and cost due to new requirements resulting from MDR are quite extensive such that some companies view them an existential threat).

159 Mateo Aboy, Timo Minssen & Effy Vayena, *Navigating the EU AI Act: Implications for Regulated Digital Medical Products*, npj Digit. Med. Sep. 6, 2024, at 1, 4, https://www.nature.com/articles/s41746-024-01232-3 [https://doi.org/10.1038/s41746-024-01232-3].

160 Calvin W. L. Ho, *Implementing the Human Right to Science in the Regulatory Governance of Artificial Intelligence in Healthcare*, 10 J.L. & Biosciences, July-Dec. 2023, at 1, 1.

161
*Id.*

162
*Id.* at 3-5.

163
*See id.* at 6, 12-13 (arguing one essential right under HRS is right of everyone, particularly patients, under risk-based approach of HRS, is right of everyone to benefit from and contribute to scientific and technological process).

164
*See id.* at 3, 24-28 (describing how HRS requires regulators to both develop inclusive networks allowing for wider participation in the development and governance of AIMDs and work toward a “common fund of knowledges” which can operate as a gap-filling technique).

165
*Id.* at 17.

166
*Id.*

167
*See id.* at 7-8 (discussing how a rights-based approach to governance of medical products includes states’ obligation to make best available applications of scientific progress to all persons, to promote scientific research, and to ensure risks involved in medical treatments are communicated transparently to patients).

168
*Id.* at 4.

169
*Id.*

170
*See* I. Glenn Cohen et al., *How AI Can Learn from the Law: Putting Humans in the Loop Only on Appeal*, 6 npj Digit. Med., Aug. 2023, at 1, 1-2, https://www.nature.com/articles/s41746-023-00906-8 [https://doi.org/10.1038/s41746-023-00906-8] (describing how person or patient “who is impacted by the AI/ML decision may offer unique insights into their case during the appeal that may be overlooked when a HITL validates every AI/ML decision”).

171
*See id.* at 2-3 (emphasizing usefulness of appeals of AI/ML decisions in contexts of scarce resource allocation and recommending appeals of AI/ML decisions should develop a consistent standard of review; a mechanism of review by specialized teams to develop general principles on given medical decisions; create mechanism to consolidate like cases; and a design where review panels have appellate discretion).

172
*Id.* at 1-2.

173 Sara Gerke et al., *The Need for a System View to Regulate Artificial Intelligence/Machine Learning-Based Software as Medical Device*, 3 npj Digit. Med., Apr. 2020, at 1, 1.

174
*Id.*

175
*Id.* at 3-4.

176
*Id.*

177
*Id.*

178
*Id.*

179 Ho, *supra* note 160, at 1.

180 Masooma Hassan et al., *Artificial Intelligence Governance Framework for Healthcare*, 38 Healthcare Mgmt. Forum, Mar. 2025, at 125, 127, https://journals.sagepub.com/doi/pdf/10.1177/08404704241291226 [https://doi.org/10.1177/08404704241291226].

181
*Id.* at 127-28.

182
*Id.* at 127.

183
*Compare id.* at 127 (describing five considerations for organizations to consider when developing and implementing AI applications, including ability to identify subject matter experts in AI ethics), *with Total Product Life Cycle for Medical Devices, supra* note 85, at 1-2 (describing utility of TPLC approach, including impact on premarket and postmarket activities).

184
*See A True Lifecycle Approach*, *supra* note 1, at 2-3.

185
*See id.* at 4

186
*Compare* Ho, *supra* note 160, at 1 (proposing a high-level human rights framework focusing on lifecycle governance), *with A True Lifecycle Approach*, *supra* note 1, at 2 (applying similar rights into more concrete checkpoints and patient-centered mechanisms across the AI lifecycle).

187
*See* Solaiman et al., *supra* note 28, at 332, 333, 339.

188
*See id.* at 335-43.

189
*See* Barry Solaiman et al., Research Guidelines for Healthcare AI Development 1, 5 (2025), [https://doi.org/10.13140/RG.2.2.10590.14402].

190
*See id.* at 4.

191
*See* Barry Solaiman, *Regulating AI-Based Medical Devices in Saudi Arabia: New Legal Paradigms in an Evolving Global Legal Order*, 16 Asian Bioethics Rev. 373, 373 (2024), https://link.springer.com/article/10.1007/s41649-024-00285-6 [https://doi.org/10.1007/s41649-024-00285-6].

192
*See id.*

193
*See A True Lifecycle Approach*, *supra* note 1, at 1.

194 Figure adapted from Figure 1 in *A True Lifecycle Approach*, *supra* note 1, at 3.

195
*See* Solaiman et al., *supra* note 189, at 2.

196
*See id.* at 4.

197
*See id.* at 5.

198
*See id.* at 1.

199
*See id.* at 8.

200
*A True Lifecycle Approach*, *supra* note 1, at 3.

201
*See* Solaiman et al., *supra* note 189, at 10.

202
*Id.* at 12.

203 These aims were articulated by the Qatar Nat’l Rsch. Fund & Qatar Genome Programme, Path Towards Precision Medicine (PPM) Call: QNRF-QGP Joint Funding Call for Proposals (CFP) Fifth Cycle 7 (2021) (unpublished call for proposals, on file with author); *see also* Qatar Nat’t Rsch. Fund & Qatar Genome Programme, Path Towards Precision Medicine (PPM) Call (4th ed. 2020) (unpublished call for proposals, on file with author).

204
*See* Solaiman et al., *supra* note 189, at 32-47.

205
*Id.* at 19-20.

206
*Id.* at 19.

207
*Id.* at 19.

208
*Id.* at 7.

209
*Id.* at 19.

210
*Id.* at 22, 44.

211
*Id.* at 19, 22, 36-38, 44.

212
*Id.* The mock website was developed internally as part of the project but not released publicly. The content of the website was based on the Guidelines Checklist found in Annex II, 32-47.

213
*See* Solaiman et al., *supra* note 189, at 5, 6.

214
*A True Lifecycle Approach*, *supra* note 1, at 4.

215
*Id.*

216 Saudi Food & Drug Auth., Guidance on Artificial Intelligence (AI) and Machine Learning (ML) Technologies Based Medical Devices 1 (2022).

217 Solaiman, *supra* note 191, at 373.

218
*Id.*

219
*Id.* at 384.

220
*Id.* at 373.

221 Saudi Food & Drug Auth., *supra* note 216, at 3.

222 Solaiman, *supra* note 191, at 379.

223 Saudi Food & Drug Auth., *supra* note 216, at 3.

224
*Id.* at 9.

225
*Id.*

226
*Id.* at 9-12.

227 Solaiman, *supra* note 191, at 383.

228 Saudi Food & Drug Auth., *supra* note 216, at 13.

229
*Id.* at 12.

230
*Id.* at 13.

231 Solaiman, *supra* note 191, at 380.

232
*Id.*

233
*Id.* at 375.

234
*Id.* at 374.

235
*Id.* at 373.

236
*Id.*

237 Saudi Food & Drug Auth., *supra* note 216, at 12.

238 Abu Dhabi Dep’t of Health, Policy on Use of Artificial Intelligence (AI) in the Healthcare Sector of the Emirate of Abu Dhabi 5 (2018), https://www.doh.gov.ae/-/media/E9C1470A575146B18015DEBE57E47F8D.ashx [https://perma.cc/HVC5-EEWG] (U.A.E.).

239 Dubai Health Auth., Artificial Intelligence in the Healthcare 2 (2021), https://www.dha.gov.ae/uploads/082022/Artificial%20Intelligence%20Policy20228457.pdf (U.A.E.).

240 For details, see Solaiman, *supra* note 191.

241 Abu Dhabi Dep’t of Health, *supra* note 238, at 10; Dubai Health Auth., *supra* note 239, § 4.11.2.

242 Abu Dhabi Dep’t of Health, *supra* note 238, at 2; Dubai Health Auth., *supra* note 239, at 1.

243 Dubai Health Auth., *supra* note 239, § 4.1.1.

244 Dubai Health Auth., *supra* note 239, §§ 4.1.2, 4.2.1.

245 Dubai Health Auth., *supra* note 239, § 4.2.3.

246 Abu Dhabi, Dep’t of Health, *supra* note 238, at 5.

247 Dubai Health Auth., *supra* note 239, § 4.3.2.

248 Dubai Health Auth., *supra* note 239, § 4.4.2.

249 Dubai Health Auth., *supra* note 239, § 4.6.

250 Dubai Health Auth., *supra* note 239, §§ 1-2.

251 Dubai Health Auth., *supra* note 239, § 4.8.

252 Dubai Health Auth., *supra* note 239, § 4.10-.11.

253 Cohen et al., *supra* note 170, at 1.

254 Solaiman, *supra* note 31, at 319-20.

255
*Bill of Rights and Responsibilities*, Primary Health Care Corp. https://www.phcc.gov.qa/patients-clients/bill-of-rights-and-responsibilities [https://perma.cc/5CBY-FSXT] (Qatar).

256 Saudi Arabia Ministry of Health, Patient Bill of Rights and Responsibilities, https://www.moh.gov.sa/en/HealthAwareness/EducationalContent/HealthTips/Documents/Patient-Bill-of-Rights-and-Responsibilities.pdf [https://perma.cc/HK7U-GJDL].

257
*Patients’ Rights and Responsibilities*, U.A.E. Gov’t Portal, https://u.ae/en/information-and-services/health-and-fitness/patients-rights-and-responsibilities [https://perma.cc/CHV8-GJES].

258
*The NHS Constitution for England*, U.K. Gov’t Dep’t Health & Soc. Care (Aug. 17, 2023), https://www.gov.uk/government/publications/the-nhs-constitution-for-england/the-nhs-constitution-for-england [https://perma.cc/PP8L-26BB].

259
*Patient Bill of Rights and Responsibilities*, U.S. Dep’t of State, https://www.state.gov/patient-bill-of-rights-and-responsibilities [https://perma.cc/8JWN-F5V8] (last visited Oct. 20, 2025).

260 Solaiman, *supra* note 31, at 328-29, 331-33.

261
*See, e.g.*, 45 C.F.R. § 46.111 (2024) (noting the criteria for IRB approval of research: informed consent, risk-benefit, privacy, and confidentiality).

262
*See* Anderson & Sutherland, *supra* note 6, at 27 (discussing the need to build capacity). *See also* World Health Org., *supra* note 18, at 32 (“[R]egulatory agencies … may not have the capacity or expertise to assess AI technologies to ensure that systematic errors do not affect diagnosis, surveillance and treatment.”).

263 On talent shortages in the GCC, see Mohammad Rashed Albous, Odeh R. Al-Jayyousi & Melodena Stephens, *AI Governance in the GCC States: A Comparative Analysis of National AI Strategies*, 82 J.A.I. Rsch. 2389, 2389 (2025), https://arxiv.org/abs/2505.02174 [https://perma.cc/KL8E-Y9GY]. *See also* Barry Solaiman, *Medical Liability in Qatar*, *in* Medical Liability in Asia and Australasia 222 (Vera Lúcia Raposo & Roy G. Beran eds., Ius Gentium vol. 94, Springer Science and Business Media B.V. 2021), https://link.springer.com/chapter/10.1007/978-981-16-4855-7_13 [https://doi.org/10.1007/978-981-16-4855-7_13] (noting the process of looking to ‘local expertise’ and ‘international best practices’ when developing law in Qatar). There is a dearth of expertise on health, law, and ethics in the country, which is reflective of the GCC more generally. *See id.*

264
*See* AI Act, *supra* note 19.

265
*See* U.S. Food & Drug Admin., *supra* note 90, at 5.

266
*See* Martin Ebers, *supra* note 4, at 3-5; *see also supra* Section 1.3.1 (discussing the WHO proposal).

267
*See supra* Section 1.3.2 (discussing FDA medical device regulations).

268
*A True Lifecycle Approach*, *supra* note 1, at 4.

269
*Id.*

270
*See* Saudi Food & Drug Auth., *supra* note 216, at 11.

271
*See* Solaiman et al., *supra* note 28, at 340.

